# Mutations in neuroligin-3 in male mice impact behavioral flexibility but not relational memory in a touchscreen test of visual transitive inference

**DOI:** 10.1186/s13229-019-0292-2

**Published:** 2019-12-02

**Authors:** Rebecca H. C. Norris, Leonid Churilov, Anthony J. Hannan, Jess Nithianantharajah

**Affiliations:** 1 Florey Institute of Neuroscience and Mental Health, University of Melbourne, 30 Royal Parade, Parkville, Victoria Australia; 2 Florey Institute of Neuroscience and Mental Health, 245 Burgundy St, Heidelberg, Victoria Australia; 3 Department of Medicine – Austin Health, Melbourne Medical School, University of Melbourne, 245 Burgundy St, Heidelberg, Victoria Australia; 4 Florey Department of Neuroscience, University of Melbourne, Parkville, Victoria Australia; 5 Department of Anatomy and Neuroscience, University of Melbourne, Parkville, Victoria Australia

**Keywords:** Autism spectrum disorder, Perseveration, Processing speed, Reaction time, Synapse

## Abstract

Cognitive dysfunction including disrupted behavioral flexibility is central to neurodevelopmental disorders such as Autism Spectrum Disorder (ASD). A cognitive measure that assesses relational memory, and the ability to flexibly assimilate and transfer learned information is transitive inference. Transitive inference is highly conserved across vertebrates and disrupted in cognitive disorders. Here, we examined how mutations in the synaptic cell-adhesion molecule neuroligin-3 (Nlgn3) that have been documented in ASD impact relational memory and behavioral flexibility. We first refined a rodent touchscreen assay to measure visual transitive inference, then assessed two mouse models of *Nlgn3* dysfunction (*Nlgn3*^−/y^ and *Nlgn3*^R451C^). Deep analysis of touchscreen behavioral data at a trial level established we could measure trajectories in flexible responding and changes in processing speed as cognitive load increased. We show that gene mutations in *Nlgn3* do not disrupt relational memory, but significantly impact flexible responding. Our study presents the first analysis of reaction times in a rodent transitive inference test, highlighting response latencies from the touchscreen system are useful indicators of processing demands or decision-making processes. These findings expand our understanding of how dysfunction of key components of synaptic signaling complexes impact distinct cognitive processes disrupted in neurodevelopmental disorders, and advance our approaches for dissecting rodent behavioral assays to provide greater insights into clinically relevant cognitive symptoms.

## Introduction

Cognitive dysfunction is a core feature of neurodevelopmental disorders. Impairments in select cognitive components such as behavioral flexibility or more flexible aspects of learning and memory such as the ability to make relational links between separate memory traces that share common elements (i.e., *generalization)* are central to neurodevelopmental disorders such as autism spectrum disorder (ASD) and schizophrenia. Behavioral flexibility refers to the capacity to modify behavior in response to changes in environmental demands [64]. One cognitive measure that assesses relational memory, and the ability to flexibly assimilate and transfer learned information is transitive inference. Transitive inference is a form of reasoning that is commonly assessed by training subjects on a hierarchy of stimulus pairs, then testing the transfer of these learned relations to novel pairs by inference from the initial training (e.g., [29, 33]). For example, subjects are trained on an overlapping series of premise stimulus pairs such that the higher member of each pair is rewarded (+) while the lower is not (−), implying a linear hierarchy in reward contingency (e.g., A^+^B^−^, B^+^C^−^, C^+^D^−^, D^+^E^−^ implies A>B>C>D>E). At test, subjects are required to infer the relations between novel pairings of these stimuli based on the training of the hierarchy (e.g., transitive pair B>D compared to the easier non-transitive pair A>E).

The capacity for transitive inference is highly conserved across vertebrates, with a large body of literature showing numerous species including humans, non-human primates, rats, mice, corvids, pigeons, and fish display transitive inference [3, 6, 14, 29, 39, 57, 58, 71, 102]. Deficits in transitive inference have been documented in brain disorders including schizophrenia [21, 100], ASD [89], attention-deficit/hyperactivity disorder [9], and Alzheimer’s disease [105]. Additionally, studies employing lesions, neuroimaging and in vivo electrophysiology have shown that functional circuitry involving the prefrontal cortex and hippocampus is essential for transitive inference in humans, non-human primates and rodents [1, 10, 27, 29, 114]. Previous rodent tests for transitive inference, predominantly in rats and some in mice, have employed discrimination of odor stimuli [26, 27, 29]. Extending these odor-based paradigms, a touchscreen test for transitive inference using visual stimuli was developed in mice [87]. Although this study involved a small sample size, the findings were promising and showcased the utility of an automated visual behavioral assay to assess transitive inference in mice that addressed some challenges associated with previous rodent paradigms (e.g., using odor as the stimulus modality, variability in hand-testing, experimenter bias).

Modeling the complex cognitive processes impacted in brain disorders using animal models is essential for elucidating the biological basis of neurodevelopmental disorders, and genetic rodent models are a predominant tool for these studies. Mutations in synapse genes are increasingly highlighted as a hub for neurodevelopmental disorders (e.g., [66]). In particular, human mutations in the neuroligin (*NLGN*) family of genes have been implicated in multiple neurodevelopmental and psychiatric disorders, with the X-linked neuroligin-3 (*NLGN3*) gene repeatedly documented in ASD [37, 50, 56, 62, 85, 91, 95, 96, 98, 109, 111–113]. Nlgn3 is a postsynaptic cell-adhesion molecule located at excitatory and inhibitory synapses, where it binds the presynaptic partner neurexin to form trans-synaptic protein complexes that govern synapse specialization, function, and plasticity [15, 48, 95]. Two mouse models have been generated to examine Nlgn3 dysfunction: (i) constitutive *Nlgn3* knock-out (*Nlgn*^*−*/*−*^) mice that have a complete loss of Nlgn3 protein expression, modeling loss-of-function mutations [97]; and (ii) *Nlgn3* arginine to cysteine point mutation knock-in (*Nlgn3*^*R451C*^) mice recapitulating the same mutation identified in two brothers with ASD [50]. The R451C mutation decreases Nlgn3 protein levels by ~90% in the forebrain, results in defective trafficking of Nlgn3 protein to the synaptic membrane and impairs binding to presynaptic neurexin [19, 22, 97]. Male *Nlgn3*^*−*/y^ and *Nlgn3*^R451C^ mice display convergent and divergent alterations in synaptic signaling and plasticity across different brain regions and cell types, thus the R451C mutation leads to loss- or gain-of-function effects at different synapses [5, 30, 31, 44, 83, 97]. Both models also display behavioral abnormalities or endophenotypes of relevance to ASD such as abnormal social and repetitive behaviors, although results across the two models have not always been consistent, nor have results across different laboratories studying the same model [18, 79, 83, 97].

Here, we aimed to investigate whether gene mutations in *Nlgn3* impact relational memory and behavioral flexibility in a test for transitive inference. We first refined and optimized a rodent touchscreen version of the transitive inference test for mice. Using our refined assay, we next assessed transitive inference in *Nlgn3*^*−*/y^ and *Nlgn3*^R451C^ mice. Deep analysis of our touchscreen behavioral data at a trial-by-trial level highlighted we could measure changes in reaction time and show that mice can transfer flexible behavioral strategies across serial discriminations. Our data show that gene mutations in *Nlgn3* do not disrupt relational memory, but significantly impact flexible responding and reaction time, suggesting perturbed processing. These results enhance our understanding of how dysfunction of key molecular players at postsynaptic signaling complexes selectively impact discrete cognitive processes and contribute to elucidating the neurobiological basis of neurodevelopmental disorders. These findings also advance our approaches in refining and dissecting rodent behavioral assays to provide greater insights into complex cognitive processes.

## Materials and methods

### Animals

Male C57BL/6J mice were purchased from the Animal Resources Centre (ARC) in Perth, Australia. One group of C57BL/6J mice (*n* = 11, 10 weeks of age) were used for the main optimization experiments, and a second group (*n* = 6, 11 weeks of age) were used to test stimulus bias. Cohorts of *Nlgn3*^R451C^ and *Nlgn3*^−/y^ mice were bred in-house from colonies established with breeding founders. *Nlgn3*^R451C^ mice (B6; 129-Nlgn3tm1Sud/J) were originally obtained from Jackson Laboratories (Bar Harbor, ME, USA). *Nlgn3*^−/y^ mice were obtained from Prof. Nils Brose (Max Planck Institute for Experimental Medicine, Göttingen Germany) generated by homologous recombination of embryonic stem cells deleting exon sequences covering the translational start site and at least 380 bp of 5’ coding sequence of the *Nlgn3* gene (described in [103] and backcrossed more than 10 generations to C57BL/6. Both *Nlgn3*^R451C^ and *Nlgn3*^−/y^ mice, and their respective WT littermate matched controls were generated by mating heterozygous females with WT males. We specifically chose to not breed male *Nlgn3*^R451C^/*Nlgn3*^−/y^ mice to minimize potential confounds, including those associated with previous reports of aggressive behavior. As *Nlgn3* is an X-linked gene, it was not possible to generate male and female homozygous null mutant mice and littermate matched WT offspring in the same litter. Additionally, the prevalence of ASD is thought to be higher in males. Therefore, we focused our analysis on male mice for the current study, for both the optimization and experimental aims.

To assess transitive inference, *Nlgn3*^R451C^ (*n* = 16) and littermate matched WT controls (*n* = 16), and *Nlgn3*^−/y^ (*n* = 15) and littermate matched WT mice (*n* = 15) were tested using two smaller cohorts (~ 6–8 mice per genotype per cohort). Four mice were excluded due to poor performance in training (see “Data analysis” section) thus final numbers analyzed were *Nlgn3*^R451C^ (*n* = 14), *Nlgn3*^−/y^ (*n* = 15), and WT controls (*n* = 29). Mice were weaned at 3–4 weeks of age and housed in mixed genotype groups of 2–4 per cage with standard rodent chow and water available *ad libitum.* Bedding consisted of sawdust chips 2 cm deep and tissue paper for nesting material. At ~10 weeks of age, mice were moved from individually ventilated cages to open top cages into humidity and temperature-controlled holding room maintained on a reversed 12:12-h light/dark cycle (lights off at 07:00). Mice were acclimatized to these conditions for a minimum of 1 week prior to handing. Pre-training began at ~ 12 weeks of age, and all behavioral testing was conducted during the dark active phase of the cycle, between 07:45 and 12:00 with the experimenter blinded to genotype during behavioral testing.

### Transitive inference touchscreen testing

#### Apparatus

Training was carried out in standard Bussey-Saksida mouse touchscreen chambers (Campden Instruments Ltd., UK) (see [45, 69, 74]. The 2-hole mask (two 7 × 7.5 cm windows separated by a 0.5 cm bar) was placed in front of the screen to minimize unintentional screen touches. Stimulus presentation and task parameters were controlled by ABET II Touch software driven using the Whisker Server Controller (Campden Instruments Ltd., UK).

#### Pre-training

Mice were first food-restricted and pre-trained as described previously [45, 69, 74]. In brief, mice were handled and weighed on three consecutive days to establish a baseline free-feeding weight, then gradually food restricted to 85–90% of free-feeding weight prior to commencing touchscreen pre-training. Mice were maintained at this restricted weight for the duration of the experiment and weighed a minimum of 5 days/week. For 2 days prior to commencing pre-training, mice were exposed to a small amount of the liquid reward (strawberry milk, Devondale 3D, Australia) in their home cages.

Mice were first pre-trained through five progressive phases for instrumental operant conditioning to learn to correctly nose-poke stimuli displayed on the touchscreen in order to obtain a liquid reward, as previously described [45, 69, 74]. Mice were required to reach a set performance criterion for each stage before advancing to the next stage. In phase 1, mice were habituated to the touchscreen chambers and to consuming the reward from the reward receptacle by being placed in the chamber for 30 min on 2 days and required to consume 200 μl of liquid reward freely available in the reward receptacle. Animals were then exposed to phase 2 or the Pavlovian stage where a single visual stimulus (black and white graphic) was displayed on the screen for 30 s, after which, the disappearance of the stimulus coincided with the presentation of a tone, illumination of the reward receptacle and delivery of the liquid reward (20 μl; criterion = complete 30 pseudorandom first-presentation trials (termed as a 'trial' from herein) within 60 min). If mice nose-poked the stimulus before 30 s had elapsed, mice were rewarded with 3 times the reward amount to encourage responding to stimuli on the screen. For phases 2–5, a trial did not advance until the reward was consumed. Mice then had to nose-poke visual stimuli that appeared on the screen to obtain a reward (phase 3, criterion = complete 30 trials within 60 min), then to initiate each new trial with a head entry into the reward receptacle (phase 4, criterion = 30 trials within 60 min). Animals then progressed onto the last pre-training phase (phase 5) designed to discourage non-selective screen responding where nose-poke responses at a blank part of the screen during stimulus presentation now produced a 5 s timeout (signaled by illumination of the house-light and no delivery of reward) (criterion = 21/30 correct responses to trials in 60 min on two consecutive days). If another response to a blank part of the screen during stimulus presentation was made, there was a 5 s inter-trial interval (ITI), and then the same trial was repeated (the same stimulus presented in the same screen location, termed a 'correction trial') until the mouse made a correct response. Therefore phases 2–5 consisted of 30 trials (pseudorandom first-presentation), and phase 5 also included an unlimited number of correction trials. Following successful pre-training, mice were moved on to training for transitive inference.

#### Transitive inference training and test

To assess and measure transitive inference, which is a complex cognitive process, we first employed a sequence of different stages of training (summarized in Table 1 and described in detail below). During the optimization and refinement of the transitive inference test, we trialed several task schedule designs (detailed in Additional file 1: Table S1). Here, we detail the final parameters established for the optimized assay which we employed to assess *Nlgn3*^−/y^ and *Nlgn3*^R451C^ mice (Table 1).

**Table 1.** Optimized design and schedule for our visual transitive inference touchscreen task in mice

Stage	Design	Trials/session	Performance criterion
Training stage 1: Stimulus exposure	Exposure to all stimulus pairs to facilitate flexibility: 1 session of each stimulus pair in order: A^+^B^−^; B^+^C^−^; C^+^D^−^; D^+^E^−^	52 trials/session; Total 4 sessions	None
Training stage 2: Premise stimulus pair learning	Learning each pairwise discrimination to criterion in order: A^+^B^−^; B^+^C^−^; C^+^D^−^; D^+^E^−^	52 trials/session; until criterion	≥ 41/52 correct (79% accuracy) in a session, plus one of the two previous or successive sessions at ≥ 34/52 correct (≥ 65% accuracy)
Training stage 3: Serial integration	All stimulus pairs presented in serial order: A^+^ B^−^; B^+^ C^−^; C^+^ D^−^; D^+^ E^−^ then sequence repeated with 13 trials of each pair presented within a session	52 trials/session; until criterion	≥ 37/52 correct (≥ 70% accuracy) in a session or max. 15 sessions (780 trials)
Training stage 4: Pseudorandom integration	All stimulus pairs (A^+^B^−^; B^+^C^−^; C^+^D-; D^+^E^−^) presented in pseudorandom order with 13 trials of each pair presented within a session	52 trials/session; until criterion	≥ 37/52 correct (≥ 70% accuracy) in a session or max. 15 sessions (780 trials)
Transitive Inference Test	20 unrewarded trials each of novel pairs AE and BD; 8 trials each of the learned pairs (A^+^B^−^, B^+^C^−^, C^+^D^−^, D^+^E^−^); all pairs presented in pseudorandom order within a session	72 trials/session; 1 session	None

Following operant pre-training, transitive inference training consisted of four stages. Each stage consisted of presentations of pairs of two equiluminescent stimuli (Fig. 2a) on the touchscreen (see [69]. Nose-poke responses to one stimulus from each pair (S+) were rewarded, coinciding with the presentation of a tone, illumination of the reward receptacle, and delivery of the liquid reward (20 μl) followed by a 20-s ITI before the next trial could commence. Responses to the other stimulus (S−) were unrewarded and triggered a 5-s time-out accompanied by illumination of the house-light, then a 5-s ITI and a correction trial, where the same trial was repeated until a correct response was made. The positional location of S+ presentation (left or right side of the touchscreen) was pseudorandom. Mice were given one daily training session (max. 60 min per session) 5–6 days a week.

Stage 1 was an introduction to the overlapping series of stimulus pairs that were intended to facilitate flexibility. It consisted of four sessions in total with one session of each stimulus pair administered in order (one session of A^+^B^−^, then B^+^C^−^, C^+^D^−^, and D^+^E^−^), and no performance criterion for advancing to the next stage. Stage 2 was stimulus pair discrimination learning that required mice to learn the discrimination of each stimulus pair in the series to a performance criterion of ≥ 79% correct responses within a session, as well as at least one of the two previous (or successive) sessions at ≥ 65% correct responses, before advancing to learn the discrimination of the next stimulus pair in the series. The criterion of 80% performance accuracy is standard for touchscreen visual discrimination learning in mice (e.g., [45]). The additional ≥65% criterion on a flanking session to ensure stable performance was employed as this is the lowest score within the 95% confidence interval (CI) for the set 80% criterion, and well outside the 95% CI for chance performance (Clopper-Pearson “exact” method). Stimulus pairs were trained in order (i.e., A^+^B^−^ then B^+^C^−^ then C^+^D^−^ then D^+^E^−^). Following successful discrimination learning of all four pairs in stage 2, mice were advanced to two integration training stages (stages 3 and 4). In stage 3 (serial integration), the four stimulus pairs were presented and then repeated in order with 13 trials of each pair presented within each session and performance criterion was ≥ 70% correct responses within a session, or a maximum of 15 sessions. Stage 4 (pseudorandom integration) was the same except the four stimulus pairs were presented in pseudorandom order with 13 trials of each pair presented within a session. In stages 1–4 correction trials were given following each incorrect response.

Once mice completed all four stages of training, animals were given one transitive inference test session which consisted of presentations of two novel pairs A>E and B>D (20 trials of each pair) and the learned premise pairs (A^+^B^−^, B^+^C^−^, C^+^D^−^, D^+^E^−^; 8 trials of each pair). Trials of the novel and learned pairs were presented in pseudorandom order and no correction trials were given. No feedback (reward, lights, tone) was given for trials of either novel pair, but correct responses to trials of previously learned premise pairs were rewarded the same way as during training.

We were also interested to measure transitive inference performance across 1–5 test sessions. Interestingly, we observed that accuracy for the transitive novel pair B>D significantly declined across sessions (main effect of session *p* = 0.004, OR = 0.946, 95% CI = 0.911, 0.983). Therefore, we decided to assess transitive inference performance in a single test session, in line with previous transitive inference protocols.

### Data analysis

Four mice (WT *n* = 2; *Nlgn3*^R451C^ *n* = 2) were excluded as outliers from all data analyses: during stage 2, 3 mice required more than 3 times the interquartile range above the upper quartile (75%) of trials to reach criterion for their genotype for any single stimulus pair, and one mouse failed to learn all four stimulus pairs. Data were collected using the ABET II Touch software and all statistical analysis was performed using STATA v14.0 (StataCorp., TX, USA).

#### Analysis of summary measures

Performance on trials (pseudorandom first-presentation) and correction trials was measured. Trials to reach criterion, errors (incorrect responses) to reach criterion and correction trials to reach criterion were collected as summary measures for each mouse for pre-training and stages 2–4 of transitive inference training.

Premise stimulus pair learning data in stage 2 violated assumptions of normality and equal variance based on visual inspection of Q-Q plots, Shapiro-WiIk tests, and Levene’s test for homogeneity of variance. We therefore used median regression to analyze significant effects of genotype, stimulus pair, and session, as well as any interaction effects, as median regression does not assume a normal distribution or homoscedasticity. Median regression models were bootstrapped x 500 to compute standard errors or had standard errors adjusted based on treating each mouse as a cluster to account for repeated measures as appropriate [67]. Median regression models the association between a set of input variables and the 50th percentile of the outcome variable and estimates differences in the median of the outcome variable between groups. Corresponding effect sizes are presented as the difference in the median values of outcome variable between groups (with corresponding 95% confidence intervals) adjusted for the selected covariates. Here, we use the term adjusted median difference (aMD) to report the effect size in median regression analyses.

Importantly, we detected no significant effects of colony or cohort across any of our analyses. Data analysis of WT littermate mice from our *Nlgn3*^R451C^ and *Nlgn3*^−/y^ colonies showed no significant effects of colony on any of our summary measures (trials, errors, or correction trials to criterion) or any of the more granular measures (odds of correct response on first-presentation or correction trials) across all four stimulus pairs during training. Therefore, WT mice from both colonies were pooled for all analyses.

#### Analysis of trial outcome

Binary outcome data (1 = correct, 0 = incorrect) were collected for each trial completed by each mouse across all training stages and the test session. Mixed-effect logistic regression models with individual mice treated as random effects were used to estimate effect sizes of genotype, stimulus pair, session, and correction vs non-correction trial on trial outcome (correct or incorrect). Logistic regression models are particularly appropriate for examining touchscreen behavioral data with binary outcomes as they allow investigation of multiple effects across several levels of clustering, e.g., subject, session, training stage, and genotype. In addition, these and related models cope well with non-normal, unbalanced (e.g., when mice reach performance criterion after completing different numbers of sessions), and heteroscedastic data. Effect sizes from these models are expressed as the factor increase/decrease in the likelihood of responding correctly (odds ratio, OR). The *p* value and 95% confidence interval (CI) are reported along with the OR to indicate the precision of the effect size estimate. An OR of 1 indicates that the respective input variable has no effect on the likelihood of a mouse responding correctly. In comparison, ORs > 1 suggest a numerically increased likelihood of a correct response, while the entire 95% CI being > 1 is indicative of a statistically significant increase in such a likelihood. Similarly, ORs < 1 suggest a numerically decreased likelihood of correct responding, and the entire 95% CI being below 1 is indicative of a statistically significant decrease.

#### Analysis of perseverative behavior

A perseveration index (PI) was calculated as per Brigman et al. [7] using the formula (total correction trials/total errors). PI assesses perseverative or repetitive incorrect responding that occurs when a mouse continues to make the same error following an incorrect response on a pseudorandom first-presentation trial: it indicates the average number of correction trials an animal requires to correct each incorrect response [8]. The lowest possible PI score is 1 reflecting a single correction trial to correct each incorrect response. PI was calculated for each mouse for each session and measured across all stages of training. PI analyses of stage 2 were restricted to sessions of each stimulus pair when data points for at least 70% of mice from each genotype were represented. Due to the variable number of sessions mice required to reach criterion on the pairs, this was 4 sessions of A^+^B^−^, 6 sessions of B^+^C^−^, 10 sessions of C^+^D^−^, and 11 sessions of D^+^E^−^. Analyses of PI across learning in stage 2 were conducted using median regression treating each animal as a cluster.

#### Assessment of TI performance

Accuracy across learned and novel pairs was assessed using median regression treating each animal as a cluster. In addition, trial outcome data for the TI test session were analyzed using mixed-effect logistic regression models as described previously. The difference from chance level performance on the novel pairs A > E and B > D was assessed using one-sample *t* tests set to a hypothesized mean of 50% accuracy.

#### Analysis of reward history

To assess whether the raw reward histories of stimuli B or D could explain the performance of TI in our study, we calculated the number of rewarded selections (Nr) and non-rewarded selections (Nn) each mouse made for each stimulus across all trials of all training stages, including correction trials. Based on previous studies, we calculated the reward/non-reward ratio (Nr/Nn) for stimuli B and D and then subtracted the ratio for B from D to assess the difference [58, 60]. In addition, we calculated the ratio of rewarded choices for B:D across all trials of training (NrB/NrD). Correlation analyses between these measures and percentage accuracy at test were performed using the Spearman rank correlation method.

#### Analysis of response and reward collection latencies

Response latencies were measured as the time taken to make a response (correct or incorrect) to a stimulus on the touchscreen following trial initiation. Correct and incorrect latencies were assessed separately as per Horner et al. [45]. For each correct response, the reward collection latency was measured as the time taken to collect the reward following the correct screen touch. Visual inspection of response latency data revealed a skewed distribution with a long right tail: latencies were > 0.8 s, mostly between 1 and 4 s, and rarely > 8 s (see Additional file 1: Figure S3). Occasional instances of extremely long latencies (up to 146 s) were recorded, but likely reflect a mouse engaging in other behaviors such as grooming before responding. To assess response latencies representing mice actively engaged in the task, we restricted the analysis to response latencies < 15 s. Latency analyses for stimulus pair learning (stage 2) were performed using data from the first 7 sessions of each stimulus pair due to some mice reaching criterion and progressing after this. Latency analyses during the final retention and integration stage (pseudorandom integration, stage 4) were performed using data from the last five sessions immediately prior to the transitive inference test session. Latency analyses were conducted using median regression treating each animal as a cluster and session as an independent variable [67].

For all our analyses at the trial level, the regression models sufficiently account for repeated measures and multiple comparisons. However, we note that a limitation is that it was not always possible to correct for multiple statistical comparison in post hoc secondary measures. We note that replication of these studies would be ideal to strengthen our conclusions; however, this is challenging given the complexity and extensive behavioral training required by such studies. It should be noted that for each experimental cohort (*Nlgn3*^R451C^ *n* = 16, WT littermate controls *n* = 16; *Nlgn3*^−/y^ *n* = 15, WT littermate controls *n* = 15), we independently trained two smaller cohorts (~ 6–8 mice per genotype per cohort) at different times, and found no significant effects of cohort on any measures, suggesting our findings are robust.

## Results

### Refining a touchscreen test for visual transitive inference in mice

To assess transitive inference in mice using the rodent touchscreen system, we designed our task based on the method previously published by Silverman et al. [87], with the aim to refine key parameters. Using C57BL/6J mice, we first trialled a 5-stimulus pair (6-term: A>B>C>D>E>F, Additional file 1: Figure S1A) version of the task that would allow multiple transitive pairs to be tested (i.e., B>D, C>E, B>E) to investigate symbolic distance effects previously described in transitive inference studies in humans [2, 12], rodents [102], and birds [6, 104]. In this first optimization experiment, we also attempted a mixed-pair training protocol for stimulus pair learning (Additional file 1: Table S1). We found that mice showed no learning or improvements in performance accuracy on any of the stimulus pairs, even after completion of 16 sessions (640 trials) of each stimulus pair (80 sessions total) (Additional file 1: Figure S1B–F). Importantly, however, we noted the perseverative index (PI, measured as the number of correction trials per incorrect response) decreased markedly after the first few sessions, indicating the mixed-pair training method rapidly promoted flexible responding even in the absence of discrimination learning (Additional file 1: Figure S1G).

**Fig. 1:**
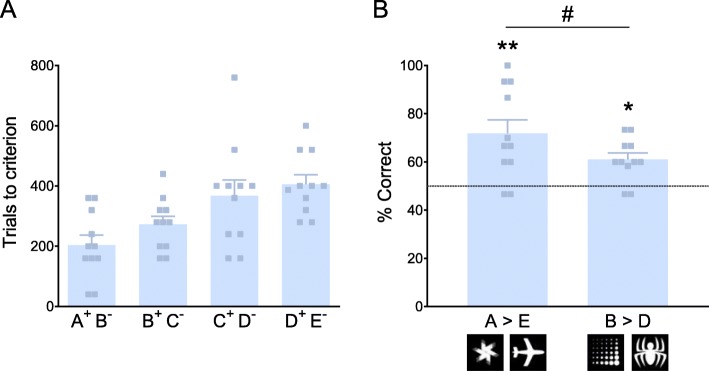
Validation of our refined visual transitive inference task. **a** Trials to criterion during learning the 4 premise stimulus pairs. **b** Performance accuracy (% correct responses) on transitive inference test session for A>E (non-transitive) and B>D (transitive). Correct performance above chance on A>E (*t* = 3.541, *p* = 0.005) and B>D (*t* = 3.016, *p* = 0.013). Accuracy on A>E greater than B>D (*t* = 1.91, *p* = 0.043). Data presented as means ± SEM. Dotted line indicates 50% accuracy (chance performance). # denotes significant difference from chance performance (^#^*p* < 0.05; ^##^*p* < 0.01); * denotes significant difference between stimulus pairs (*p < 0.05)

We next trialed a serial stimulus pair training protocol consistent with Silverman et al. [87] in our second optimization experiment, in which mice were given sessions of a single pair until they reached the performance criterion and then advanced to the next pair in the series. Following successful discrimination learning of all five stimulus pairs, we tested mice on two integration stages (Additional file 1: Table S1). Here, we found that although mice were able to learn the discrimination of the five premise pairs when trained serially (Additional file 1: Figure S1H), none of the mice were able to complete the serial integration stage (Additional file 1 Table S1), even after 35 sessions (1400 trials) of training, highlighting the complexity of a 6-term visual discrimination task in mice. Therefore, in our third and last optimisation experiment, we further modified the training protocol to adopt a 4-stimulus pair (5-term) task using the serial pair learning protocol established in our previous experiment with modifications to performance criteria for the serial and pseudorandom integration stages (see “Materials and methods” section). Using this refined protocol, we were successfully able to train mice to discriminate the four premise pairs (Fig. 1a), then complete two stages of integrated stimulus pair presentations (serial then pseudorandom) before being tested for transitive inference. The transitive inference test session contained trials of the novel stimulus pairs A>E (non-transitive) and B>D (transitive), as well as the previously learned pairs (A^+^B^−^, B^+^C^−^, C^+^D^−^, and D^+^E^−^). We observed that C57BL/6J mice showed discrimination for both the non-transitive pair A>E and the transitive pair B>D above chance level (Fig. 1b), consistent with previously published transitive inference performance in animal models. Moreover, discrimination accuracy for A>E was greater than for B>D as expected.

**Fig. 2:**
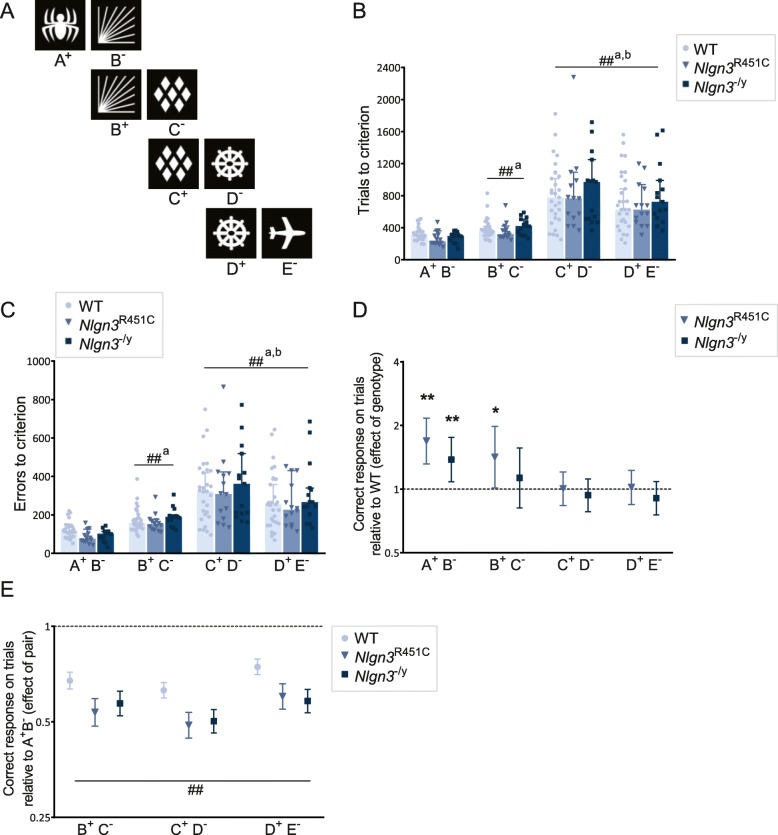
*Nlgn3*^−/y^ and *Nlgn3*^R451C^ mice display normal premise stimulus pair learning. **a** Optimized visual stimuli used for premise stimulus pairs. **b** Trials and **c** errors to criterion during discrimination learning for the 4 premise stimulus pairs (stage 2). Data presented as medians ± 95% CI. **b** Mice required more trials to reach criterion on C^+^D^−^ and D^+^E^−^ than A^+^B^−^ (C^+^D^−^: *p* < 0.001, aMD = 488.552, 95% CI = 370.371, 606.732; D^+^E^−^: *p* < 0.001, aMD = 407.035, 95% CI = 288.854, 525.215). **c** Mice made more errors to criterion on pairs B^+^C^−^, C^+^D^−^, and D^+^E^−^ than A^+^B^−^ (B^+^C^−^: *p* = 0.038, aMD = 51.241, 95% CI = 2.94, 99.540; C^+^D^−^: *p* < 0.001, aMD = 203.035, 95% CI = 154.735, 251.334; D^+^E^−^: *p* < 0.001, aMD = 153.379, 95% CI = 105.080, 201.679). **d** Effect of genotype on the likelihood of responding correctly to trials (pseudorandom first-presentation) during premise pair learning relative to WT (represented by dotted line; 1 = no difference to WT). A^+^B^−^ (*Nlgn3*^R451C^: *p* < 0.001, OR = 1.69, 95% CI = 1.314, 2.165; *Nlgn3*^−/y^: *p* = 0.009, OR = 1.379, 95% CI = 1.083, 1.755). B^+^C^−^ (*Nlgn3*^R451C^ *p* = 0.042, OR = 1.416, 95% CI = 1.013, 1.979). **e** Effect of stimulus pair on the likelihood of responding correctly to trials during premise pair learning relative to A^+^B^−^ (represented by dotted line; 1 = no difference to A^+^B^−^). See Additional file 1: Table S3 for statistics. # denotes significant difference between stimulus pairs (^#^*p* < 0.05; ^##^*p* < 0.01). * denotes significant difference between genotypes (*p < 0.05, **p < 0.01)

It is well understood that stimulus bias can have major impacts on learning and flexible responding to visual touchscreen tasks. In our first optimization experiment, we indeed found suggestions of bias for some stimuli (performance on the first training session of each stimulus pair, Additional file 1: Figure S2A). Therefore, we trialed two new stimuli (flash and wheel) and tested discrimination learning in a separate naive cohort of mice to confirm no differences in stimulus preference (Additional file 1: Figure S2B). The flash and wheel stimuli were therefore incorporated as part of the optimized stimulus set for our final protocol (Fig. 2a). To further validate this chosen stimulus set, analysis of WT mice performance on the first exposure to each stimulus pair (stage 1) in our final experiments described below showed no bias for pairs A^+^B^−^, C^+^D^−^, or D^+^E^−^ (Additional file 1: Figure S2C). For the second pair B^+^C^−^, however, mice were a little less likely than chance to select stimulus B. This could be interpreted as a bias towards stimulus C or against B, but we reasoned this was unlikely to be the case as we do not see similar biases on pairs sharing those stimuli (A^+^B^-^ or C^+^D^-^). Therefore, this observed pattern more likely reflects some learning within that first A^+^B^−^ session which had the least ambiguity in stimulus-reward contingencies, relative to the exposure of subsequent pairs. Additionally, stimulus C (diamonds) in our optimal set displayed no indication of bias when assessed in our first optimization experiment (stimulus F, Additional file 1: Figure S2A).

### Normal operant and stimulus pair learning in Nlgn3^R451C^ and Nlgn3^−/y^ mice

Having optimized our refined touchscreen test for transitive inference, we next wanted to assess our two models, *Nlgn3*^R451C^ and *Nlgn3*^−/y^ mice, on this task. All rodent touchscreen tests involve a behavioral shaping phase to pre-train naive mice through several operant learning stages to accurately nose-poke stimuli displayed on the touchscreen to obtain a reward. Both *Nlgn3*^R451C^ and *Nlgn3*^−/y^ mice were able to complete the pre-training phases at the same rate as WT littermate-matched controls, indicating mutations in *Nlgn3* do not impact simple operant learning (Additional file 1: Table 2).

We then trained *Nlgn3*^R451C^ and *Nlgn3*^−/y^ mice on our optimized transitive inference task consisting of four stages (Table 1), where they were first trained to discriminate each stimulus pair in order (A^+^B^−^, B^+^C^−^, C^+^D^−^, D^+^E^−^) (stages 1 and 2). We observed that both *Nlgn3* mutant mouse models required similar numbers of trials compared to WT mice to reach the learning criterion on the premise pairs (Fig. 2b). Although we found no statistically significant differences between genotypes in the trials taken to learn each stimulus pair, there was a significant difference in the trials all mice required to learn the different stimulus pairs, with pairs B^+^C^−^, C^+^D^−^, and D^+^E^-^ being harder to learn (more trials to acquire discrimination) compared to the first pair A^+^B^−^. Additionally, mice required more trials to learn the final two pairs C^+^D^-^ and D^+^E^−^ compared to B^+^C^−^. A similar pattern was observed for the number of incorrect responses or errors to reach the learning criterion for the four stimulus pairs; there were no genotype differences between *Nlgn3*^R451C^, *Nlgn3*^−/y^, and WT mice; however, all mice made significantly more errors to learn pair B^+^C^−^ compared to pair A^+^B^−^, and more errors to learn C^+^D^−^ and D^+^E^−^ than B^+^C^−^ (Fig. 2c). In contrast to the trials to reach the learning criterion, mice made fewer errors learning the final pair D^+^E^−^ compared to pair C^+^D^−^. Collectively, these data indicate that mice find learning additional overlapping stimulus pairs more challenging as the number of stimulus pairs increased, suggesting a progressive increase in cognitive load as each pair was acquired.

We extended the data analysis of stimulus pair learning (stage 2) to more deeply examine performance at the level of each trial (pseudorandom first-presentation) using logistic regression to measure (i) the likelihood of *Nlgn3*^R451C^ and *Nlgn3*^−/y^ mice making a correct response compared to WT mice (effect of genotype) and (ii) the likelihood of mice making a correct response on subsequent stimulus pairs relative to the first pair A^+^B^−^ (effect of stimulus pair). There was a significant pair x genotype interaction (*p* < 0.001) on the likelihood of making a correct response, so we assessed differences between genotypes for each stimulus pair separately. For pair A^+^B^−^, both *Nlgn3*^R451C^ and *Nlgn3*^−/y^ mice were significantly more likely to correctly respond than WT mice, indicating enhanced accuracy during discrimination learning of the first pair (Fig. 2d). For the second pair B^+^C^−^, *Nlgn3*^R451C^ mice were still more likely to respond accurately than WT mice, but this was not observed for *Nlgn3*^−/y^ mice. In comparison, for the latter stimulus pairs C^+^D^−^ and D^+^E^−^ that required more trials to learn the discrimination, there were no differences between genotypes on the likelihood of correct responding. The small but significant increase in accurate responding for *Nlgn3*^R451C^ and *Nlgn3*^−/y^ mice evident in this analysis highlights the increased sensitivity gained from using a trial level measure to detect differences in accuracy compared to the summary measure of total trials required to reach a learning criterion. In addition to the genotype effects, there were significant effects of stimulus pair on the odds of correct responding for all genotypes: mice were less likely to make a correct response in trials while learning pairs B^+^C^−^, C^+^D^−^, and D^+^E^−^ compared to pair A^+^B^−^ (Fig. 2E, Additional file 1: Table S3), indicating the likelihood of making a correct response reduced as task load increased, consistent with our findings on the trials taken to learn stimulus pairs.

### Mutations in Nlgn3 impact flexible responding

**Fig. 3.**
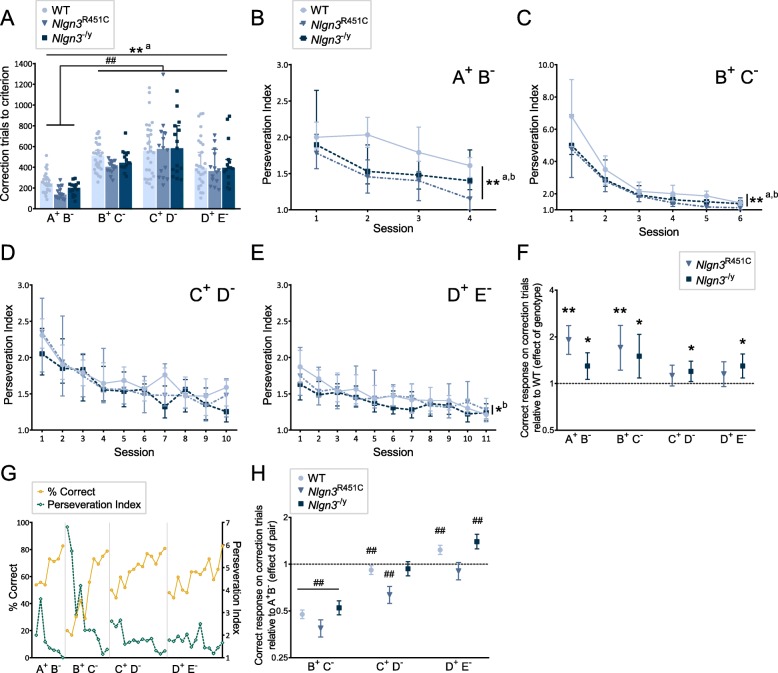
*Nlgn3*^−/y^ and *Nlgn3*^R451C^ mice show altered behavioral flexibility and perseverative behavior. **a** Total correction trials to criterion during discrimination learning for the 4 premise stimulus pairs (stage 2). *Nlgn3*^R451C^: *p* = 0.024, aMD = − 75, 95%, CI = − 139.88, − 10.13; *Nlgn3*^−/y^: *p* = 0.752, aMD = − 12, 95%, CI = − 86.85, 62.85. Relative to A^+^B^−^ (B^+^C^−^: *p* < 0.001, aMD = 257, 95%, CI = 217.025, 296.975; C^+^D^−^: *p* < 0.001, aMD = 368, 95% CI = 256.102, 479.899; D^+^E^−^: *p* < 0.001, aMD = 180, 95% CI = 125.529, 234.471. Data presented as medians ± 95% CI. Perseverative index in stage 2 for **b** A^+^B^−^, **c** B^+^C^−^, **d** C^+^D^−^, and D^+^E^−^. A^+^B^−^ (*Nlgn3*^R451C^: *p* < 0.001, aMD = − 0.425, 95% CI = − 0.605, − 0.246; *Nlgn3*^−/y^: *p* = 0.001, aMD = − 0.231, 95% CI = − 0.366, − 0.096). B^+^C^−^ (*Nlgn3*^R451C^: *p* = 0.001, aMD = − 0.708, 95% CI = − 1.112, − 0.304; *Nlgn3*^−/y^: *p* = 0.010, aMD = − 0.472, 95% CI = − 0.828, − 0.116. D^+^E^−^ (*Nlgn3*^−/y^: *p* = 0.029, aMD = − 0.097, 95% CI = − 0.184, − 0.009). Data presented as median ± 95% CI. **a–e** Median regression treating each animal as a cluster, and genotype and session as independent variables. **f** Effect of genotype on the likelihood of responding correctly to correction trials during stimulus pair learning relative to WT mice (represented by dotted line; 1 = no difference to WT). A^+^B^−^ (*Nlgn3*^R451C^: *p* < 0.001, OR = 1.909, 95% CI = 1.3542, 2.364; *Nlgn3*^−/y^: *p* = 0.010, OR = 1.296, 95% CI = 1.064, 1.578). B^+^C^−^ (*Nlgn3*^R451C^: *p* = 0.002, OR = 1.701, 95% CI = 1.220, 2.371; *Nlgn3*^−/y^: *p* = 0.014, OR = 1.500, 95% CI = 1.085, 2.074). C^+^D^−^ (*Nlgn3*^−/y^: *p* = 0.020, OR = 1.197, 95% CI = 1.029, 1.393). D^+^E^−^ (*Nlgn3*^−/y^: *p* = 0.004, OR = 1.298, 95% CI = 1.086, 1.551). **g** Representative plot of % correct response (grey points plotted on left *Y* axis) and perseveration index (black dotted points plotted on right *Y* axis) for every session for each premise pair (stage 2). Data represent raw values from one WT mouse. **h** Effect of pair on the likelihood of responding correctly to correction trials relative to A^+^B^−^ during premise pair learning (represented by dotted line, 1 = no difference to pair A^+^B^−^). See Additional file 1: Table S5 for statistics. **f**–**h** Data presented as odds ratio ± 95% CI, logistic regression with individual animals as random effects and session as independent variable. # denotes significant difference between stimulus pairs (^#^*p* < 0.05; ^##^*p* < 0.01). * denotes significant difference between genotypes (*p < 0.05, **p < 0.01). a = *Nlgn3*^R451C^ significantly different to WT, b = *Nlgn3*^−/y^ significantly different to WT

During stimulus pair learning, when mice made an incorrect response to a trial (pseudorandom first-presentation), a correction procedure (correction trial) followed whereby the same trial was repeated until a correct choice was made. Correction trials are used in most touchscreen learning tests to counteract potential stimulus position bias, ensure animals receive a consistent number of rewards per session and facilitate learning [16, 45]. When we assessed the total number of correction trials animals made to reach the learning criterion across stimulus pairs, we found *Nlgn3*^R451C^ mice required marginally fewer correction trials compared to WT mice (Fig. 3A), but this was not observed in *Nlgn3*^−/y^ mice. There was also a significant difference between stimulus pairs, with all mice making significantly more correction errors learning stimulus pairs B^+^C^−^, C^+^D^−^, and D^+^E^−^ compared to the first stimulus pair A^+^B^−^ (Fig. 3A).

To measure flexible responding, a perseveration index (PI) (correction trials/incorrect responses) was calculated for each mouse per session to provide a dynamic measure of perseverative incorrect responding independent of trials taken to reach the learning criterion on each stimulus pair. We found significant interaction effects between genotype, stimulus pair, and session on PI (*Nlgn3*^R451C^ × pair, *p* < 0.001; *Nlgn3*^−/y^ × pair, *p* = 0.026; pair × session, *p* < 0.001). We therefore analyzed the effects of genotype and session on PI for each stimulus pair separately using median regression. All mice became less perseverative as training sessions progressed on each pair (Additional file 1: Table S4) but mutations in *Nlgn3* significantly altered perseverative responding. Strikingly, both *Nlgn3*^R451C^ and *Nlgn3*^−/y^ mice had significantly lower PIs compared to WT mice for the early stimulus pairs A^+^B^−^ and B^+^C^−^ (Fig. 3B, C) where sensitivity for detection in PI changes is the highest. There were no significant differences between genotypes for C^+^D^−^ (Fig. 3D) but *Nlgn3*^−/y^ mice continued to show lower PIs for D^+^E^−^ (Fig. 3E).

Investigating the data more deeply, we also examined perseveration at the level of each trial using logistic regression to analyze the likelihood of making a correct response to correction trials, where a higher odds ratio (OR) value indicates a greater likelihood of a correct choice and therefore reduced perseveration. There was a significant stimulus pair × genotype interaction (*p* < 0.001), so we analyzed the data by genotype and stimulus pair separately. *Nlgn3*^R451C^ and *Nlgn3*^−/y^ mice were more likely to respond correctly to correction trials on stimulus pairs A^+^B^−^ and B^+^C^−^ than WT mice (Fig. 3G), consistent with our PI results of decreased perseverative responding. *Nlgn3*^−/y^ mice remained more likely to respond accurately to correction trials on stimulus pairs C^+^D^−^ and D^+^E^−^, however, this was not significant for *Nlgn3*^R451C^ mice.

One might expect that PI would be inversely linked to accuracy; as animals learn and respond more accurately to the rewarded stimulus (S+) across training sessions, their PI scores would decline, reflecting reduced perseverative responding to the unrewarded stimulus (S−). Our data for the first two stimulus pairs are consistent with this idea: accurate responding increased while perseverative responding decreased as animals learned the A^+^B^−^ discrimination, and there was a marked drop in accuracy concurrent with a sharp increase in PI when animals transitioned from A^+^B^−^ to B^+^C^−^ (Fig. 3G). However, examining the subsequent transitions in learning the latter stimulus pairs revealed a dissociation between accuracy and PI. While performance accuracy continued to drop to ~ 50% when animals transitioned to pairs C^+^D^−^ and D^+^E^−^, the concurrent increases in PI were modest and continued to reduce in magnitude compared to that observed with B^+^C^−^. This resulted in a progressive reduction in PI across pairs B^+^C^−^ to D^+^E^−^ observed across all genotypes (Fig. 3B–E). This pattern suggests that mice not only show greater flexible responding with increased training on one stimulus pair discrimination but that they are able to transfer this flexible behavior when challenged with having to subsequently learn new overlapping discriminations.

To assess the ability to transfer flexible behavior, we measured the likelihood of making correct responses on correction trials (thus inversely representing the likelihood of perseverative incorrect responding) on latter pairs relative to A^+^B^-^. This analysis revealed insightful trajectories displayed by all mice (irrespective of genotype) which were strongly impacted by stimulus pair order, in line with the pattern we observed in PI. As shown in Fig. 3H (data in Additional file 1: Table S5), the likelihood of correctly responding to correction trials was strikingly low for pair B^+^C^−^ relative to A^+^B^−^ or any other pair for all genotypes, indicating a dramatic increase in perseverative responding for pair B^+^C^−^ relative to A^+^B^−^. This was followed by an upward trajectory in the likelihood of correctly responding to correction trials. This likelihood increased for pair C^+^D^−^ to be similar to pair A^+^B^−^, although WT and *Nlgn3*^R451C^ mice remained more perseverative on pair C^+^D^−^ compared to A^+^B^−^. Subsequently, by the time mice transitioned to learning D^+^E^−^, the likelihood of correctly responding to correction trials was greater relative to the first pair A^+^B^−^ for WT and *Nlgn3*^−/y^ mice. These results indicate that (i) perseverative incorrect responding decreased for each subsequent discrimination from pairs B^+^C^−^ to D^+^E^−^, and (ii) the rate of this decline was so significant that mice showed greater flexibility in adaptive responding when learning the final stimulus pair D^+^E^-^ than the first A^+^B^−^ pair discrimination. Importantly, a similar upward trajectory in the likelihood of correct responding across pairs B^+^C^−^ to D^+^E^−^ was not evident for pseudorandom first-presentation trials (Fig. 2E), highlighting that this effect was specific to perseveration (correction trials). These findings in PI and correction trials are in line with our results from the optimization experiments showing increased flexible responding can occur independently of improvements in discrimination learning performance accuracy, further supporting our approach to incorporate presentations of all stimulus pairs early in training to promote flexibility (stage 1, see Additional file 1: Figure S4). Additionally, the progressive increase in flexible responding with increased training likely contributes to why differences between genotypes were most evident during the discrimination learning of the early stimulus pairs; these represent a more sensitive window to detect changes in flexibility in comparison to the latter stimulus pair discriminations when mice converge towards an optimal level of correction trial performance with minimal perseveration. Collectively, these findings support that acquiring a “rule” or response strategy that promotes behavioral flexibility can be generalized to benefit subsequent performance under overlapping conditions.

### Nlgn3 mutations do not impact transitive inference and relational memory

**Fig. 4:**
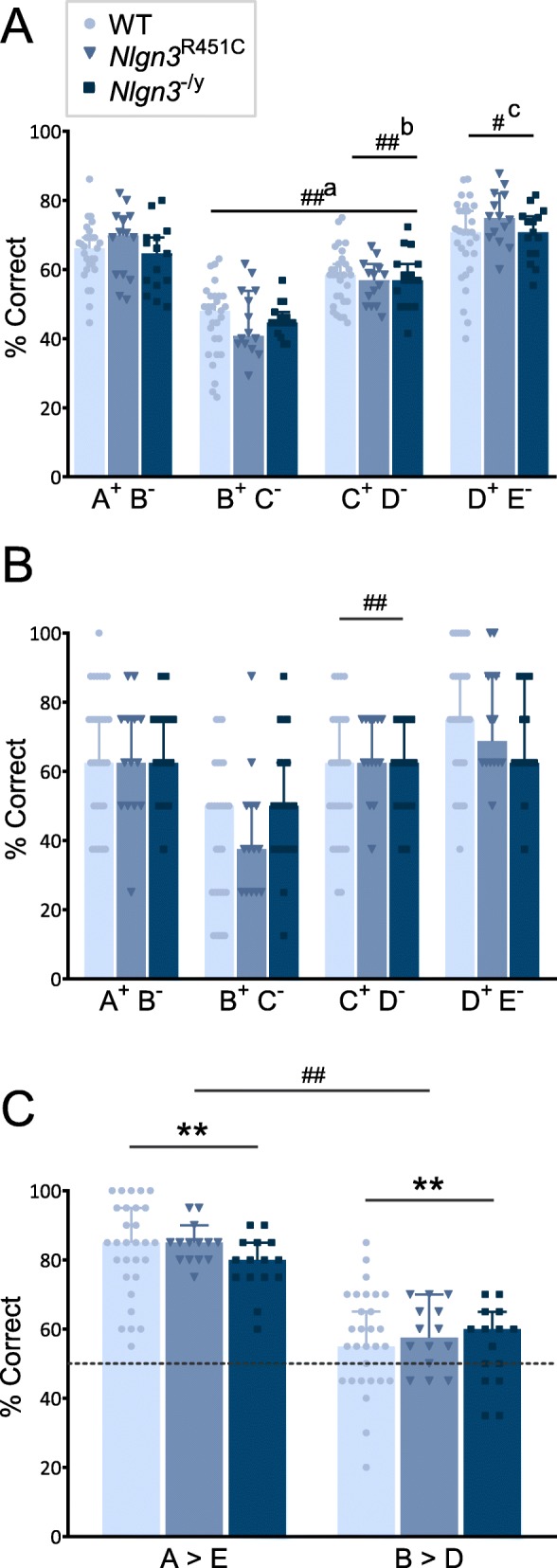
Stimulus pair retention and transitive inference is intact in *Nlgn3*^−/y^ and *Nlgn3*^R451C^ mice. Performance accuracy (% correct responses) on the 4 premise pairs during **a** pseudorandom integration (stage 4, last 5 sessions) and **b** transitive inference test session. **a** Relative to A^+^B^−^, B^+^C^−^: *p* < 0.001, aMD = − 0.205, 95% CI = − 0.254, − 0.156; C^+^D^−^: *p* < 0.001, aMD = − 0.082, 95% CI = − 0.121, − 0.043). C^+^D^−^ greater than B^+^C^−^ (*p* < 0.001, aMD = 0.123, 95% CI = 0.082, 0.165. D^+^E^−^ marginally higher than A^+^B^−^ (*p* = 0.043, aMD = 0.045, 95% CI = 0.001, 0.088. B B^+^C^−^ compared to all other pairs (A^+^B^−^: *p* = 0.007, aMD = − 0.25, 95% CI = − 0.432, − 0.068; C^+^D^−^: *p* = 0.002, aMD = − 0.25, 95% CI = − 0.410, − 0.090; D^+^E^−^: *p* < 0.001, aMD = − 0.375, 95% CI = − 0.582, − 0.168). Median regression with genotype and stimulus pair as independent variables, and each animal treated as a cluster. **b** See Additional file 1: Table S7 for statistics. **c** Accuracy on A>E (non-transitive) and B>D (transitive) during transitive inference test (dotted line indicates chance performance; AE>chance *t*_(57)_ = 22.44, *p* < 0.001; BD>chance *t*_(57)_ = 3.98, *p* < 0.001). Data points plotted for each mouse, data represent median ± 95% CI. # denotes significant difference between stimulus pairs (^#^*p* < 0.05, ^##^*p* < 0.01, a = lower than A^+^B^−^, b = higher than B^+^C^−^, c = higher than A^+^B^-^); * denotes significant difference from chance (50% accuracy) (*p < 0.05, **p < 0.01)

Following discrimination learning of the four premise pairs of stimuli (stages 1 and 2), mice were exposed to two further integration training stages where all stimulus pairs were presented in serial order (stage 3) and then pseudorandom (stage 4) within a session until a performance criterion was reached. Both *Nlgn3*^R451C^ and *Nlgn3*^−/y^ mice showed no differences in performance as measured by the trials taken to complete these stages (Additional file 1: Table S6). To assess retention of the learned pairs, we analyzed performance accuracy on all stimulus pairs during the last five sessions of the pseudorandom integration stage (stage 4) prior to being tested for transitive inference. We identified no statistically significant effect of genotype on performance accuracy, however, all mice were more accurate on the end stimuli pairs (A^+^B^−^ and D^+^E^−^) compared to the middle pairs (B^+^C^−^ and C^+^D^−^), resulting in the characteristic “U-shaped” pattern of accuracy for the premise pairs (Fig. 4A) indicative of the serial position effect described in previous transitive inference studies [26, 29]. Median regression analysis indicated accuracy on the middle stimulus pairs (B^+^C^−^ and C^+^D^−^) was significantly lower than for the end pairs (A^+^B^−^ and D^+^E^−^), accuracy on C^+^D^−^ was significantly greater than B^+^C^−^, and performance accuracy on the last stimulus pair D^+^E^−^ was marginally higher than the first stimulus pair A^+^B^−^.

We next administered the test session to assess transitive inference. This contained trials of novel stimulus pairs A>E (non-transitive) and B>D (transitive), as well as previously learned premise pairs (A^+^B^−^, B^+^C^−^, C^+^D^−^, D^+^E^−^). Median regression analysis of performance accuracy on the four premise pairs revealed all mice displayed a “U-shaped” serial position curve pattern, like that observed during the pseudorandom integration stage (Fig. 4B) with no significant differences between genotypes or interaction effects identified. Like the preceding pseudorandom integration stage, mice were less accurate on stimulus pair B^+^C^−^ compared to all other pairs. We did note the transitive inference test session results were more variable, most likely due to the smaller number of trials available for analysis from this single test session compared to the five sessions used for the retention analysis on the pseudorandom integration stage. We extended the transitive inference test session analysis using logistic regression to measure the likelihood of accurate responding at the trial level for each stimulus pair. We again found no differences between genotypes nor any genotype × stimulus pair interaction but observed significant effects of stimulus pair. All mice were most likely to correctly respond to trials of pair D^+^E^−^, then A^+^B^−^, compared to the two middle pairs (B^+^C^−^ and C^+^D^−^) (see Additional file 1: Table S7 for statistics). In line with performance accuracy, mice were least likely to respond correctly to trials of pair B^+^C^−^ compared to all other stimulus pairs (Fig. 4B).

Performance analysis on the novel non-transitive pair A>E and the novel transitive pair B>D revealed no significant differences between genotypes, indicating *Nlgn3*^R451C^ and *Nlgn3*^−/y^ mice display normal relational memory and are able to perform transitive inference. All mice showed the expected pattern of greater discrimination accuracy on the non-transitive pair A>E compared to the transitive pair B>D (Fig. 4C, median regression *p* < 0.001, adjusted median difference (aMD) = 0.25, 95% CI = 0.213, 0.287), with performance accuracy on both pairs above chance level. Further analysis using logistic regression to examine the likelihood of accurate responding at the trial level showed the same pattern where all mice were more likely to make a correct response to the non-transitive pair A>E compared to the transitive pair B>D (*p* < 0.001, OR = 0.271, 95% CI = 0.224, 0.329).

Several explanations of transitive inference have been proposed in which accurate selection of B>D can be due to stimuli B and D acquiring differing associative values over many trials of training due to the specific reinforcement history of the stimuli [33, 34, 108]. Simple reinforcement-based explanations for the choice of B>D include that rewarded responses to B^+^ and D^+^ may be unequal, or an animal may make fewer unrewarded responses to B^−^ (in the context of A^+^ B^−^) than to D^−^ (in the context of C^+^D^−^), due to the unambiguous reward association of A^+^ [108]. Simple measures of reinforcement history alone have been shown to be insufficient to account for transitive inference performance across the variety of training and testing conditions in which it occurs [58, 59, 92], although more complex reinforcement-based models make predictions that align well with animal performance (e.g., [34]). An experiment involving extended training on D^+^E^−^ intended to boost positive association of D^+^ above B^+^ showed animals were still able to correctly select B>D following this intervention, suggesting mechanisms other than raw reinforcement history contribute to transitive inference performance [60]. Based on the method by Lazareva and colleagues (2012), we calculated the ratio of rewarded choices for B:D, as well as the difference between the ratio of rewarded to non-rewarded selections between stimuli B and D, based on all trials across all stages of training. We found no significant correlation between either of these and performance on pair B>D in the transitive inference test session, suggesting differences in reinforcement history overall do not account for the selection of B>D by mice in our study (data not shown).

Performance of transitive inference has also been suggested to correlate with memory for the learned premise pairs, and indeed several studies have shown this for accuracy on pair B>D [12, 24, 63]. To assess whether this was the case in our study, we examined whether performance accuracy on the learned pairs could explain performance accuracy on the novel pairs B>D or A>E in the test session. In contrast to previous work, we found no significant correlation between accuracy on the learned pairs and accuracy on pair B>D for mice in our study (data not shown). We did, however, observe a small but significant correlation between accuracy on the learned premise pairs and accuracy on the non-transitive pair A>E (*r*_*s*_ = 0.270, *p* = 0.040).

### Processing speed is altered in Nlgn3^R451C^ and Nlgn3^−/y^ mice

In addition to measuring performance accuracy (correct vs incorrect responding), we were interested to examine in-depth the latency measures captured during responding in our touchscreen test. Reaction times are often assessed in human cognitive tests and show changes with increased processing demands of the response [41, 81]. Several latency measures can be calculated in the rodent touchscreen system, including latency to respond (time taken to make a correct or incorrect response following stimulus presentation) and reward collection latency (time taken to collect reward following a correct response).

**Fig. 5:**
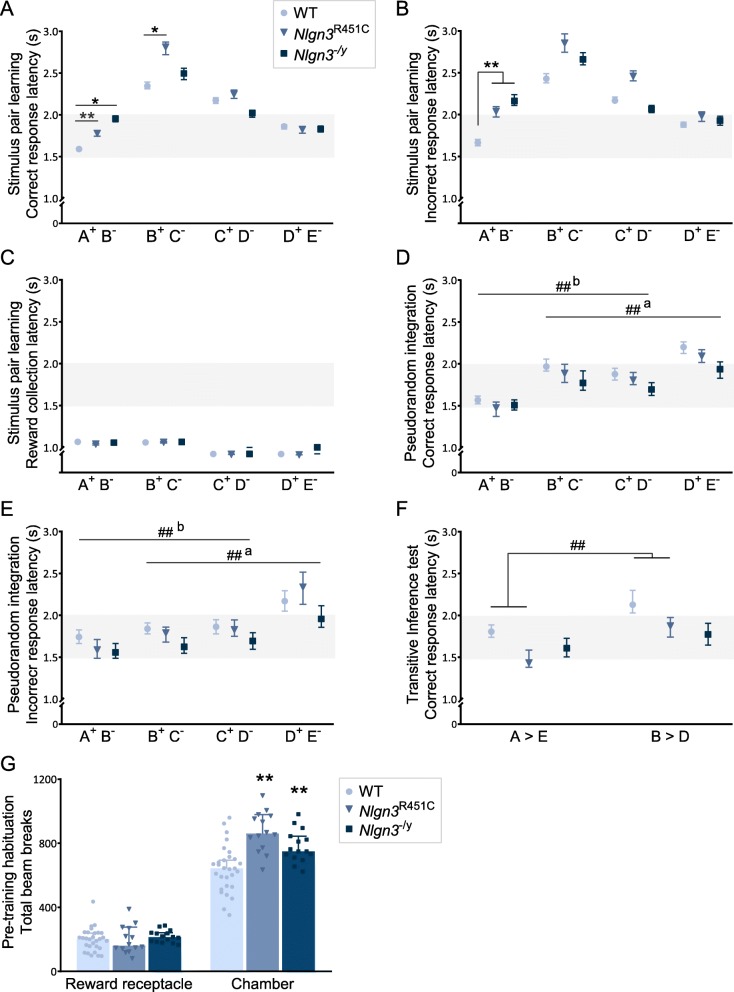
Response and reward collection latencies in *Nlgn3*^*R451C*^ and *Nlgn3*^−*/y*^ mice. **a** Correct and **b** incorrect response latencies, and **c** reward collection latencies discrimination learning for the 4 premise stimulus pairs (stage 2). **a**–**b** See Additional file 1: Table S8 for statistics. **d** Correct and **e** incorrect response latencies during pseudorandom integration (stage 4, last 5 sessions). See Additional file 1: Table S9 for statistics. **F** Correct response latencies for A>E (non-transitive) and B>D (transitive) during transitive inference test session (WT: *p* = 0.002, aMD = 0.324, 95% CI = 0.120, 0.528; *Nlgn3*^R451C^: *p* < 0.001, aMD = 0.441, 95% CI = 0.253, 0.629; *Nlgn3*^−/y^: *p* = 0.065, aMD = 0.165, 95% CI = − 0.010, 0.340). **a**–**f** Data presented as median ± 95% CI. **g** Total infrared beam breaks in the reward receptacle and chamber (front and back beams) during habituation to the touchscreen chambers in pre-training. One-way ANOVA *F*_(2, 60)_ = 16.17, *p* < 0.01; post hoc Bonferroni correction for multiple comparisons; WT to *Nlgn3*^R451C^ *p* < 0.001, WT to *Nlgn3*^−/y^ *p* = 0.006). Data presented as mean ± SEM. * denotes significant differences between genotypes (*p < 0.05; ** *p* < 0.01); # denotes significant differences between pairs (^##^*p* < 0.01, ^#^*p* < 0.05); a = difference from pair A^+^B^−^, b = difference from pair D^+^E^−^. Horizontal grey bands added to aid visual comparison

We first focused on analyzing correct and incorrect response latencies to trials (pseudorandom first-presentation) during stimulus pair learning (stage 2). We observed multiple interaction effects (*p* = 0.015 for pair × *Nlgn3*^−/y^ genotype; *p* = 0.002 for pair × correct response); therefore, employed median regression analysis to assess the effect of genotype on response latencies for each stimulus pair separately. When learning to discriminate the first stimulus pair A^+^B^−^, both *Nlgn3*^R451C^ and *Nlgn3*^−/y^ mice took significantly longer to make correct (Fig. 5A) and incorrect (Fig. 5B) responses compared to WT mice (Additional file 1: Table S8A). *Nlgn3*^R451C^ mice continued to take longer to make correct responses while learning the next stimulus pair B^+^C^−^, but not incorrect responses. In comparison, for stimulus pair B^+^C^−^, *Nlgn3*^−/y^ mice showed no significant differences in response latencies to either correct or incorrect choices. Similarly, we did not find significant differences in response latencies between genotypes while learning stimulus pairs C^+^D^−^ or D^+^E^−^.

This data also revealed a convex pattern in response latencies as mice progressed through learning the stimulus pairs, similar to an inverted serial position curve, suggesting that response latencies were dynamically impacted by the transitions to each new stimulus pair, and may therefore reflect the difficulty or demands on cognitive processing associated with learning a series of overlapping discriminations (Fig. 5A, B). All mice took significantly longer to make correct and incorrect choices for pair B^+^C^−^ compared to the first pair A^+^B^−^ (Additional file 1: Table S8B). This behavior persisted for WT mice as they learned subsequent pairs, where both correct and incorrect response latencies were longer for pairs C^+^D^−^ and D^+^E^−^ than for A^+^B^−^. Similarly, *Nlgn3*^R451C^ mice took longer to make correct and incorrect choices on the latter pairs C^+^D^−^, and on correct (but not incorrect) responses for pair D^+^E^−^ than for A^+^B^−^. In contrast, *Nlgn3*^−/y^ mice had similar response latencies (correct and incorrect) on stimulus pairs C^+^D^−^ and D^+^E^−^ relative to pair A^+^B^−^, possibly because their initial response latencies while learning the first pair were significantly longer than WT mice, and thus their response latencies became similar to WT mice as they learned successive pairs. In contrast to the dynamic changes in response latencies, reward collection latencies during stimulus pair learning remained relatively stable, with no significant differences between genotypes (Fig. 5C). These data highlight the specificity of the observed changes in response latencies in *Nlgn3*^R451C^ and *Nlgn3*^−/y^ mice and strongly suggest the changes in reaction time are not simply driven by a general change in locomotor speed or activity.

Extending this analysis, we were interested to understand whether there might be differences between response latencies during the final pseudorandom integration stage (stage 4) compared to initial pair learning (stage 2). We were also interested to know whether latencies during stage 4 would correlate inversely with accuracy, which might be expected if more difficult discriminations required greater processing. We therefore assessed correct and incorrect response latencies during the last five sessions of pseudorandom integration training (stage 4). Regression analyses did not identify differences in response latencies between genotypes for any of the stimulus pairs when analyzed separately, in contrast to our findings for stimulus pair discrimination learning (stage 2). However, similar to stage 2, all mice took longer to respond to pair B^+^C^−^ and the latter pairs (C^+^D^−^, D^+^E^−^) compared to A^+^B, but the inverted “U-shaped” pattern was absent. Correct response latencies were similar for pairs B^+^C and C^+^D^−^ and longest for pair D^+^E^−^ compared to all other pairs (Fig. 5D, Additional file 1: Table S9A) despite performance accuracy on D^+^E^−^ being the highest during pseudorandom integration training (Fig. 4A). A similar, but less pronounced, pattern was observed for incorrect responses (Fig. 5e, Additional file 1: Table S9B). These results show that the requirement to integrate multiple overlapping pairs in the same session and/or extended training impacts response latencies in our transitive inference task. While we observed that response reaction times during initial learning of the stimulus pairs were inversely correlated with performance accuracy and differentially impacted by *Nlgn3* mutations, this pattern was no longer evident later on during the final sessions of integration training.

Lastly, when we examined response latencies during transitive inference testing, we observed WT and *Nlgn3*^R451C^ mice had significantly longer response latencies for the more difficult transitive pair B>D compared to the easier non-transitive pair A>E, with *Nlgn3*^−/y^ mice displaying a similar trend (*p* = 0.065) (Fig. 5F). These data are consistent with a correlation between response latencies and cognitive load or processing demands during the transitive inference test session, which aligns with similar findings in other tasks [94, 106].

### *Nlgn3*^R451C^ and *Nlgn3*^−/y^ mice display hyperactivity during habituation to the touchscreen chambers

Previous studies have reported hyperactivity in both *Nlgn3*^R451C^ and *Nlgn3*^−/Y^ mice [52, 79, 83]. As motor function can impact performance in cognitive assays, we assessed spontaneous locomotor activity when we first exposed mice to the touchscreen chambers during the habituation pre-training stage. Locomotor and exploratory activity were measured by infrared beam breaks at the front and back of the chambers, and nose-pokes made inside the reward receptacle. There were no differences in the number of exploratory nose-pokes into the reward receptacle between WT, *Nlgn3*^R451C^, and *Nlgn3*^−/y^ mice (Fig. 5G). However, both *Nlgn3*^R451C^ and *Nlgn3*^−/y^ mice displayed greater numbers of beam breaks within the chamber (front and back) compared to WT mice (Fig. 5G), consistent with the hyperactive phenotype previously reported in both *Nlgn3* mutant models. These data are particularly interesting given that *Nlgn3*^R451C^ and *Nlgn3*^−/y^ mice display an opposing *slower* response latency phenotype during our task training. Importantly, these results further highlight the specificity of the observed changes in response latencies in *Nlgn3*^R451C^ and *Nlgn3*^*−/y*^ mice during our task acquisition and suggest this may reflect changes associated with cognitive load and processing speed.

## Limitations

Similar to that previously reported [87], our refined protocol still requires lengthy training due to its complexity and appears to be more difficult for mice to acquire compared to odor-based transitive inference paradigms, which might be expected given the ethological priority of olfaction over vision as a sense in mice. We were also unable to successfully develop a more complex version of the visual transitive inference test for mice (5-pair, 6-term) which may have allowed a greater dissection of measures such as the symbolic distance effect commonly assessed in human and non-human primate tests of transitive inference [2, 68]. A general consideration facing rodent transitive inference tasks is whether the same mechanisms underlie performance across species, and we discuss this in more detail in the conclusion section below. Lastly, we did not assess female *Nlgn3*^R451C^ and *Nlgn3*^−/−^ mice due to the different and therefore extended breeding strategy that would have been required to generate cohorts of both male and female mutant mice and respective WT littermates. Although ASD is currently thought to be more prevalent in males, the importance of assessing both male and female mice in disease models is critical.

## Conclusions

This study represents the most comprehensive analysis of a visual transitive inference task in rodents, and the first to assess transitive inference in two mouse models with mutations in the same disease-relevant gene. We first established a refined task to assess transitive inference in mice using visual stimuli in the rodent touchscreen system. We show that C57BL/6J mice can acquire our task and accurately discriminate the transitive stimulus pair B>D, and the non-transitive pair A>E, in line with previous literature. Employing our refined task, we found that both *Nlgn3*^R451C^ and *Nlgn3*^−/y^ mice and their WT littermates exhibit normal transitive inference. We were able to show that all mice displayed the “U-shaped” serial position curve pattern of discrimination accuracy, with greater accurate responding on the end stimulus pairs (A^+^B^−^ and D^+^E^−^) compared to the middle pairs (B^+^C^−^ and C^+^D^−^), consistent with previous transitive inference work in humans (e.g., [32]) and rodents employing odor-based paradigms [25, 27, 29]. *Nlgn3*^R451C^ and *Nlgn3*^−/y^ mice took longer to make response choices alongside more accurate responding during discrimination learning of early premise stimulus pairs and showed consistently reduced perseveration across learning the premise pairs. Our data is the first to dissect response latency measurements in a rodent transitive inference test, showing dynamic changes in response latencies during transitions in overlapping pairwise discrimination learning and differential demands in cognitive load between transitive and non-transitive novel discriminations. Together, these results suggest reaction times in rodent touchscreen tasks can be utilized to measure cognitive demands or processing speed. Our results on this adapted version of transitive inference confirm our task can robustly measure visual discrimination learning of hierarchical, overlapping stimulus pairs, relational memory, and behavioral flexibility in mice.

Relational memory was intact in both our mouse models with gene mutations in *Nlgn3*. There is currently no available cognitive data measuring transitive inference in individuals with mutations in *NLGN3.* For the two brothers with the *NLGN3* R451C point mutation, clinical notes reveal the eldest has autism with intellectual disability and the younger brother autism with Asperger’s syndrome [47, 50] highlighting the importance of genetic background on phenotypic variability. Lesion studies indicate transitive inference critically depends on the hippocampus and the prefrontal cortex in rodents [26, 27, 101]. It is well undertood that both *Nlgn3*^R451C^ and *Nlgn3*^−/y^ mice show disrupted hippocampal synaptic transmission and plasticity [30, 31, 44]. Despite these hippocampal signaling changes, the capacity for transitive inference was not impacted in *Nlgn3* mutant mice in our study. In line with this, others have shown performance on other hippocampal-dependent tests such as contextual fear conditioning and spatial learning in the Morris water maze is preserved or even enhanced in *Nlgn3*^R451C^ and *Nlgn3*^−/y^ mice [18, 52, 79, 97]. *Nlgn3* mutant mice also show divergent subregion, circuit, and cell-type-specific changes in synaptic plasticity across the brain [30, 31, 83], leading to the proposal that the precise role of *Nlgn3* (and other *Nlgn* isoforms) depends on the specific molecular environment of the synapse investigated [78]. This also highlights the complexity in unraveling how mutations in synapse genes essential for the development of distinct circuits can lead to the region and cell-type-specific changes in synaptic signaling and plasticity, thereby impacting the modulation of distinct cognitive behaviors at the systems level.

In contrast to relational memory, both *Nlgn3*^R451C^ and *Nlgn3*^−/y^ mice robustly displayed altered behavioral flexibility in the form of decreased perseverative responding, possibly indicative of greater responsiveness to negative feedback. This was particularly evident during the first discrimination (stimulus pair A^+^B^−^), and the first overlapping discrimination that required value or state/context updating (stimulus pair B^+^C^−^). Our data allowed in-depth analysis of responding when animals made repeated errors in correction trials. This kind of perseverative incorrect responding indicates lose-stay behavior, compared to switching to make a correct response or lose-shift behavior. The proportion of lose-shift to lose-stay behavior provides an indication of how well subjects integrate negative feedback to guide future choices. The lower PI scores of *Nlgn3*^R451C^ and *Nlgn3*^−/y^ mice indicate they displayed greater lose-shift behavior, potentially implicating *Nlgn3* to be key in the prefrontal-striatal-brainstem circuits shown to mediate value-updating processes that drive lose-shift over lose-stay strategies [4, 20, 40, 70]. The observed convergent reductions in perseverative responding in both *Nlgn3*^R451C^ and *Nlgn3*^−/y^ mice aligns with previous work showing both models display enhanced motor learning on a rotarod, used as a proxy for the acquisition of a repetitive motor routine [83].

Human mutations in *NLGN3* have been documented in ASD [37, 50, 56, 62, 85, 91, 95, 96, 98, 109, 111–113]. ASD is typically described as involving increased repetitive behaviors and impaired flexibility. Contrary to this, our results show that *Nlgn3* mutations in mice *decreased* perseveration and *enhanced* behavioral flexibility. Our data may reflect a *Nlgn3* model-specific effect on flexible behavior. It is important to note symptom profiles in ASD can be highly heterogeneous, with evidence that individuals with ASD exhibit *impaired*, *preserved*, and *enhanced* cognitive flexibility [36, 43, 55, 75, 76, 88]. Although there is conceptual overlap between cognitive flexibility (assessed using performance-based tests) and the repetitive and restricted behaviors reported in ASD (from self- or carer-reports), these two measures do not always strongly correlate [23, 61, 65, 93, 110] because they may differentially engage the processes underlying flexibility. Flexibility is a broad term that is used to encompass dissociable cognitive processes such as inhibitory control, value-updating, action-outcome updating, and task-switching which can be assessed in different ways which may further contribute to the mixed reports across ASD studies [64].

Our analysis also revealed insights into decision-making strategies in mice and evidence for unlearned response bias. We observed that upon first exposure to stimuli pairs (stage 1), mice displayed high perseverative responding in that although accuracy and win-stay behavior were at chance level for first-presentation trials, mice were more likely than chance to repeat their previously incorrect responses (Additional file 1: Figure S4). During serial pair-learning (stage 2), mice showed progressively smaller impacts to perseverative responding during transitions to each new stimulus pair, resulting in mice being less perseverative while learning the final stimulus pair (D^+^E^−^) compared to the first pair (A^+^B^−^). When considering our mice required more trials to learn the latter stimulus pairs in the series, this suggests that when learning overlapping discriminations, mice retain and transfer a rule or response strategy that promotes lose-shift responding, while this is not the case for win-stay behavior. This is consistent with evidence that win-stay probabilities do not change across multiple reversals in stimulus-reward contingency, while lose-shift probabilities increased with more reversals [51]. In serial reversal studies, animals require fewer trials and errors to reach criterion across successive reversals, suggesting they acquire a form of structural learning that reversals (a change in context) can occur [17, 49]. In our study, mice required *more* trials to learn the latter stimulus pairs, highlighting the greater complexity of integrating overlapping stimulus pairs relative to serial reversal learning. Despite this, the smaller dips in accuracy with transitions to the latter stimulus pairs (C^+^D^−^ and D^+^E^−^) compared to the first (A^+^B^−^ to B^+^C^−^) suggest a similar kind of learning might have occurred.

Another striking difference was alterations specifically in response latencies (but not reward collection) during early learning, where *Nlgn3*^R451C^ and *Nlgn3*^−/y^ mice took longer to make correct and incorrect choices on pair A^+^B^−^. These response-specific latency changes suggest alterations in speed of processing or decision-making especially because the physical distance animals must travel to complete a response or collect reward is identical; therefore, latency differences between these two actions are unlikely to be due to motoric reasons. Additionally, *Nlgn3* mutant mice were also more accurate on pair A^+^B^−^, which could indicate a difference in speed-accuracy trade-off, a concept demonstrated across several rodent studies [11, 80, 82, 84]. Further studies explicitly examining this would be needed to address this [42]. Response latency measurements were also dynamically impacted by transitions in learning the different stimulus pairs and appeared to be positively correlated with cognitive load or processing demands. Similarly, during the transitive inference test, mice exhibited longer response times during the more difficult transitive discrimination B>D compared to the easier non-transitive discrimination A>E. These findings are consistent with human cognitive literature where reaction times increase when demands on information processing are greater, including when inference is required compared to a simpler comparison (e.g., [81, 106]). Our results are also consistent with human data and drift-diffusion models which show reaction times are inversely correlated with differences in value between items [72]. Our study presents the first detailed analysis of response latencies in a rodent test of transitive inference and indicates response latencies from the touchscreen system are useful indicators of processing demands or decision-making processes. Future work incorporating video analysis of trial behavior during testing will provide greater depth and understanding of the response choice behavior of animals.

Human neuroimaging studies demonstrate that transitive inference activates the hippocampus [41, 106, 114] and prefrontal-parietal networks [1, 38]. In vivo electrophysiology in non-human primates reveals dorsolateral prefrontal cortex neurons respond selectively when transitive judgments are performed, independent of the reward value of the stimuli [10]. In rodents, lesion studies suggest similar brain regions, namely, the hippocampus and prefrontal cortex are important for transitive inference [26, 27, 101]. Additionally, connectivity between these two regions (involving perirhinal, entorhinal, fornix) appears to be crucial for the expression of transitive inference in rodents [29] and monkeys [14]. Although these same brain regions have been identified to be critical for transitive inference in rodents, non-human primates, and humans, a key point of consideration facing rodent transitive inference tasks is whether the same mechanisms underlie performance across species [73, 99]. A related unknown is whether mice can use transitive reasoning to infer dimensional, *explicitly* transitive relationships (e.g., size) without extended training. Humans and non-human primates can employ similar cognitive mechanisms in feedback-trained transitive inference tasks to those used to evaluate explicitly transitive relationships [2, 12, 53, 99]. An ethologically relevant example for mice is social dominance, where mice form consistent linear dominance hierarchies that exhibit triangle transitivity [107]. However, whether mice can infer relative rank through observation of social interactions alone (i.e., perform transitive inference in a social dominance context), which has been shown in other species [39, 77] is unknown.

In human transitive inference studies, reaction times remain stable for learned end pairs but increase across training for pairs not containing end stimuli [2], which is thought to reflect the employment of a cognitive strategy integrating the middle pairs into a mental schema [41]. It is currently unclear whether rodents are also able to employ a similar “mental schema” in transitive inference [102]. Although this question is beyond the scope of our study, our data show response latencies were faster for the middle pairs B^+^C^−^ and C^+^D^−^ during the final integration stage than during initial pair learning, which is more consistent with an effect of extended training than integration of pairs into a mental schema. In other rodent touchscreen tests, response latencies typically decrease as training progresses (e.g., [28, 46, 54]). We also note that our finding of higher accuracy on pair D^+^E^−^ than A^+^B^−^ at the end of training aligns with predictions of a reinforcement-based account of transitive inference that does not rely on a mental schema [34]. Other alternative explanations for the choice of B>D in transitive inference tasks include the acquisition of “select” vs “avoid” rules for stimuli and the related idea of rule “stacks” that prioritize actions based on some stimuli over others [13, 35].

Our study extends the work by Silverman et al. [87] who showed a touchscreen-based transitive inference assay employed to test BTBR mice (which have been used to model ASD-relevant phenotypes) could detect impaired discrimination for the non-transitive pair A>E, but intact discrimination for the transitive pair B>D when compared to C57BL/6J mice. While this study was promising, few animals were tested. Our work has refined and validated this assay to show that transitive inference can be assessed using a visual touchscreen-based test. Our refined assay combined with the deep data analysis approach provides a sensitive tool to measure complex stimulus pair learning, integration, relational memory, perseveration, flexibility, and reaction times in mice. Early research suggested children with ASD perform similarly to neurotypical controls on a transitive inference test that manipulated the length of stimuli (an explicitly transitive property) [86]. There are two contemporary reports assessing transitive inference using visual stimuli in ASD led by the same group [89, 90]. One study reported adults with ASD exhibit impaired accuracy on the non-transitive pair A>E [89], while the other study reported no change in transitive inference performance in adolescents with ASD [90] which is in line with our findings. It is recognized that cognitive processes such as the capacity to learn and remember elements of different experiences, flexibly compare and reintegrate these memories to allow generalization of learning, are impacted in ASD. Therefore, future studies expanding the assessment of transitive inference in larger cohorts with ASD would be informative to gain more comprehensive understandings about the profile of cognitive symptoms in neurodevelopmental disorders.

In summary, we show that our refined touchscreen test for transitive inference robustly measures visual discrimination learning of hierarchical, overlapping stimulus pairs and relational memory, behavioral flexibility, and reaction times in mice. Our findings in two mouse models of *Nlgn3* dysfunction expand our understanding of how specific gene mutations that disrupt synaptic signaling complexes can lead to selective alterations in distinct cognitive processes, of relevance to unraveling the neurobiological basis of neurodevelopmental disorders including ASD.

## Supplementary information

**Additional file 1: Figure S1.** Task design optimisation to refine the transitive inference task. **Figure S2.** Assessment of stimulus bias to optimise the premise stimulus pairs. **Figure S3.** Histogram of the skewed distribution of response latencies (with a long tail) during stimulus pair learning (Stage 2). **Figure S4.** Percentage of trials that were Win-stay (win-stay/win-stay + win-shift) and Lose-shift (lose-shift/lose-shift + lose-stay) for the 4 premise stimulus pairs across all genotypes during the first 4 sessions of training (Stage 1). **Table S1.** Summary of optimisation Experiments 1-3 in C57BL/6J mice to refine the transitive inference touchscreen task. **Table S2.** Sessions to criterion on pre-training stages to acquire operant learning. **Table S3.** Effect of stimulus pair on the likelihood of responding correctly to trials (pseudorandom first-presentation) relative to pair A+B- during premise pair learning (Stage 2). **Table S4.** Effect of session on Perseveration Index (PI) for all genotypes across all pairs during premise pair learning (Stage 2). **Table S5.** Effect of stimulus pair on the likelihood of responding correctly to correction trials relative to pair A+ B- during premise pair learning (Stage 2). **Table S6.** Trials to criterion on the serial (Stage 3) and pseudorandom (Stage 4) integration training stages. **Table S7.** Likelihood of responding correctly on the 4 learned premise pairs (relative to A+B- or D+E-) on the transitive inference test. **Table S8.** Correct and incorrect response latencies to trials (pseudorandom first-presentation) during premise stimulus pair learning (Stage 2) relative to WT and stimulus pair A+B-. **Table S9.** Differences between correct and incorrect response latencies for the 4 premise stimulus pairs (relative to A+B-, B+C- or D+E-) for all genotypes during pseudorandom integration (Stage 4, last 5 sessions of training).

## Data Availability

The datasets generated in the current study are available from the corresponding author on reasonable request.
