# Supplementary material for: Mutations in neuroligin-3 in male mice impact behavioral flexibility but not relational memory in a touchscreen test of visual transitive inference
Source: Mol Autism. 2019 Dec 2;10:42. doi: 10.1186/s13229-019-0292-2 (PMC6889473; doi:10.1186/s13229-019-0292-2)
Supplement: Supplementary file 1 — Additional file 1: Figure S1. Task design optimisation to refine the transitive inference task. Figure S2. Assessment of stimulus bias to optimise the premise stimulus pairs. Figure S3. Histogram of the skewed distribution of response latencies (with a long tail) during stimulus pair learning (Stage 2). Figure S4. Percentage of trials that were Win-stay (win-stay/win-stay + win-shift) and Lose-shift (lose-shift/lose-shift + lose-stay) for the 4 premise stimulus pairs across all genotypes during the first 4 sessions of training (Stage 1). Table S1. Summary of optimisation Experiments 1-3 in C57BL/6J mice to refine the transitive inference touchscreen task. Table S2. Sessions to criterion on pre-training stages to acquire operant learning. Table S3. Effect of stimulus pair on the likelihood of responding correctly to trials (pseudorandom first-presentation) relative to pair A+B- during premise pair learning (Stage 2). Table S4. Effect of session on Perseveration Index (PI) for all genotypes across all pairs during premise pair learning (Stage 2). Table S5. Effect of stimulus pair on the likelihood of responding correctly to correction trials relative to pair A+ B- during premise pair learning (Stage 2). Table S6. Trials to criterion on the serial (Stage 3) and pseudorandom (Stage 4) integration training stages. Table S7. Likelihood of responding correctly on the 4 learned premise pairs (relative to A+B- or D+E-) on the transitive inference test. Table S8. Correct and incorrect response latencies to trials (pseudorandom first-presentation) during premise stimulus pair learning (Stage 2) relative to WT and stimulus pair A+B-. Table S9. Differences between correct and incorrect response latencies for the 4 premise stimulus pairs (relative to A+B-, B+C- or D+E-) for all genotypes during pseudorandom integration (Stage 4, last 5 sessions of training). [file 13229_2019_292_MOESM1_ESM.pdf]

SUPPLEMENTARY INFORMATION

Mutations in neuroligin-3 in male mice impact behavioral flexibility but not relational memory in a touchscreen test of visual transitive inference

Rebecca HC Norris[1], Leonid Churilov[2,3], Anthony J Hannan[1,4,5], Jess Nithianantharajah[1,4].

1. Florey Institute of Neuroscience and Mental Health, University of Melbourne, 30 Royal Parade, Parkville Victoria Australia.
2. Florey Institute of Neuroscience and Mental Health, 245 Burgundy St, Heidelberg, Victoria Australia.
3. Department of Medicine – Austin Health, Melbourne Medical School, University of Melbourne, 245 Burgundy St, Heidelberg, Victoria Australia.
4. Florey Department of Neuroscience, University of Melbourne, Parkville Victoria, Australia.
5. Department of Anatomy and Neuroscience, University of Melbourne, Parkville, Victoria, Australia

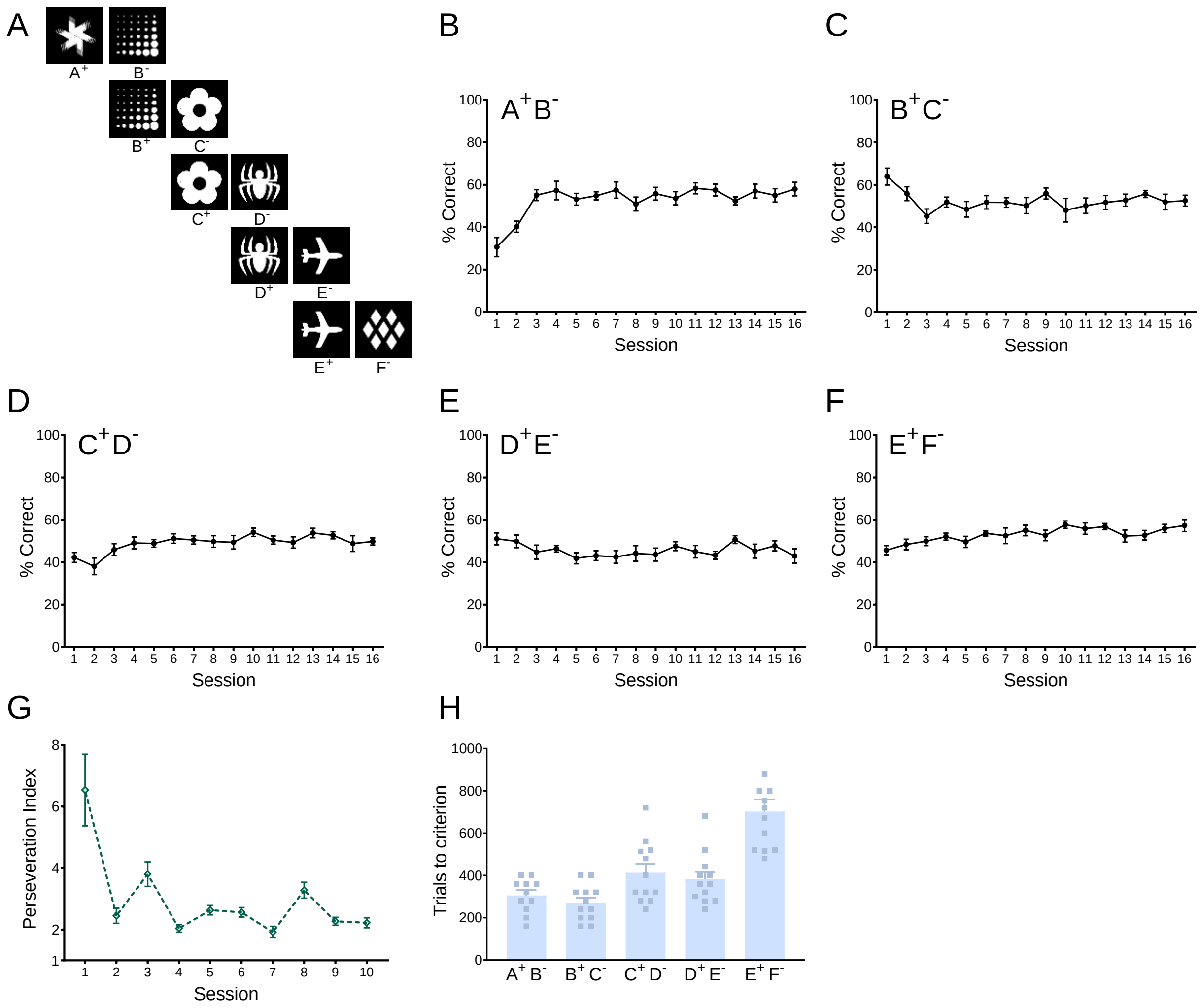

Supplementary Figure 1: Task design optimisation to refine the transitive inference task. (A) Stimulus pairs (5-pair, 6-term) tested in optimisation Experiments 1 and 2. (B-F) Tracking performance (% correct response) across sessions during stimulus pair training using the mixed-block design in Experiment 1. Statistics (one-way repeated measures ANOVAs with Sidak's correction for multiple comparisons): A^+B^- ($F_{6.71} = 7.26$, $p < 0.001$); B^+C^- ($F_{5.67} = 1.69$, $p = 0.144$); C^+D^-($F_{5.31} = 3.17$, $p = 0.061$); D^+E^- ($F_{5.94} = 1.12$, $p = 0.892$); E^+F^- ($F_{5.26} = 2.09$, $p = 0.332$). Significance in pair A^+B^- likely due to overcoming stimulus bias in the first two sessions as no improvement evident from sessions 3 – 16 ($F_{6.13} = 0.831$, $p = 0.553$). (G) Decrease in Perseveration Index in Experiment 1. (H) Trials to criterion when mice were trained sequentially on the 5 stimulus pairs in Experiment 2. Data presented as mean ± SEM, n = 11 C57BL/6J mice.

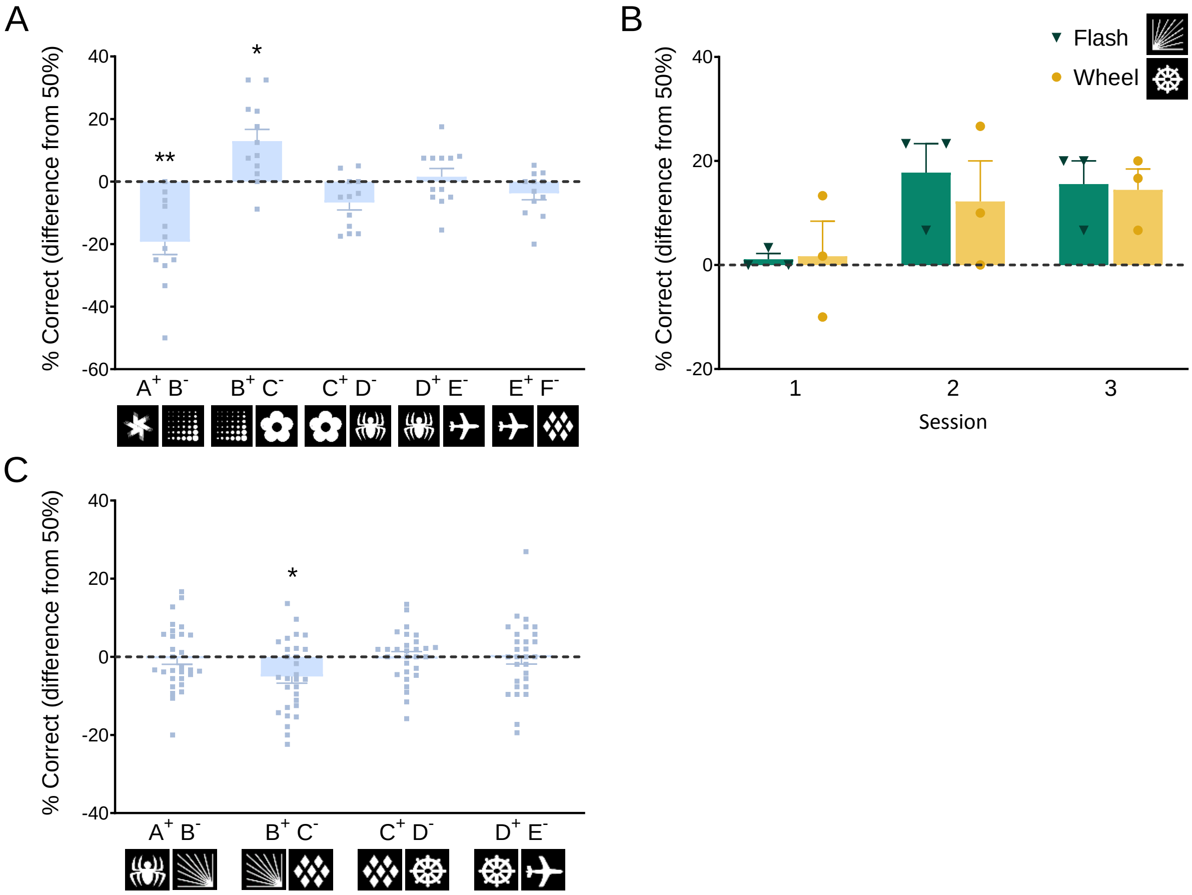

Supplementary Figure 2: Assessment of stimulus bias to optimise the premise stimulus pairs. (A) Performance (% correct responses) on the first session of each stimulus pair in Experiment 1 (n = 11 C57BL/6J mice). On trials of A^+B^-, mice showed a bias to avoid A and/or select B ($t = 4.68$, $p = 0.003$). On trials of pair B^+C^-, mice again showed a bias to select B and/or avoid C ($t = 3.46$, $p = 0.026$). On trials of C^+D^-, mice showed a suggestive bias to avoid C and/or select D, though this was not significant. Stimulus pairs D^+E^- and E^+F^- showed no bias. One sample t-tests with Sidak's correction for multiple comparisons. (B) No bias evident across three sessions of testing for new flash and wheel stimuli using a naive cohort of C57BL/6J mice (n = 6). (C) Performance (% correct responses) of WT mice (n = 29) on the first session of each stimulus pair (Stage 1) of our final experiment. Mice performed at chance level for pairs A^+B^-, C^+D^- and D^+E^- but 5.04% below chance on pair B^+C^- ($t = 2.96$, $p = 0.025$). Performance expressed as divergence from chance (50% accuracy), bars show means ± SEM, * denotes significant difference from chance (*$p < 0.05$, **$p < 0.01$).

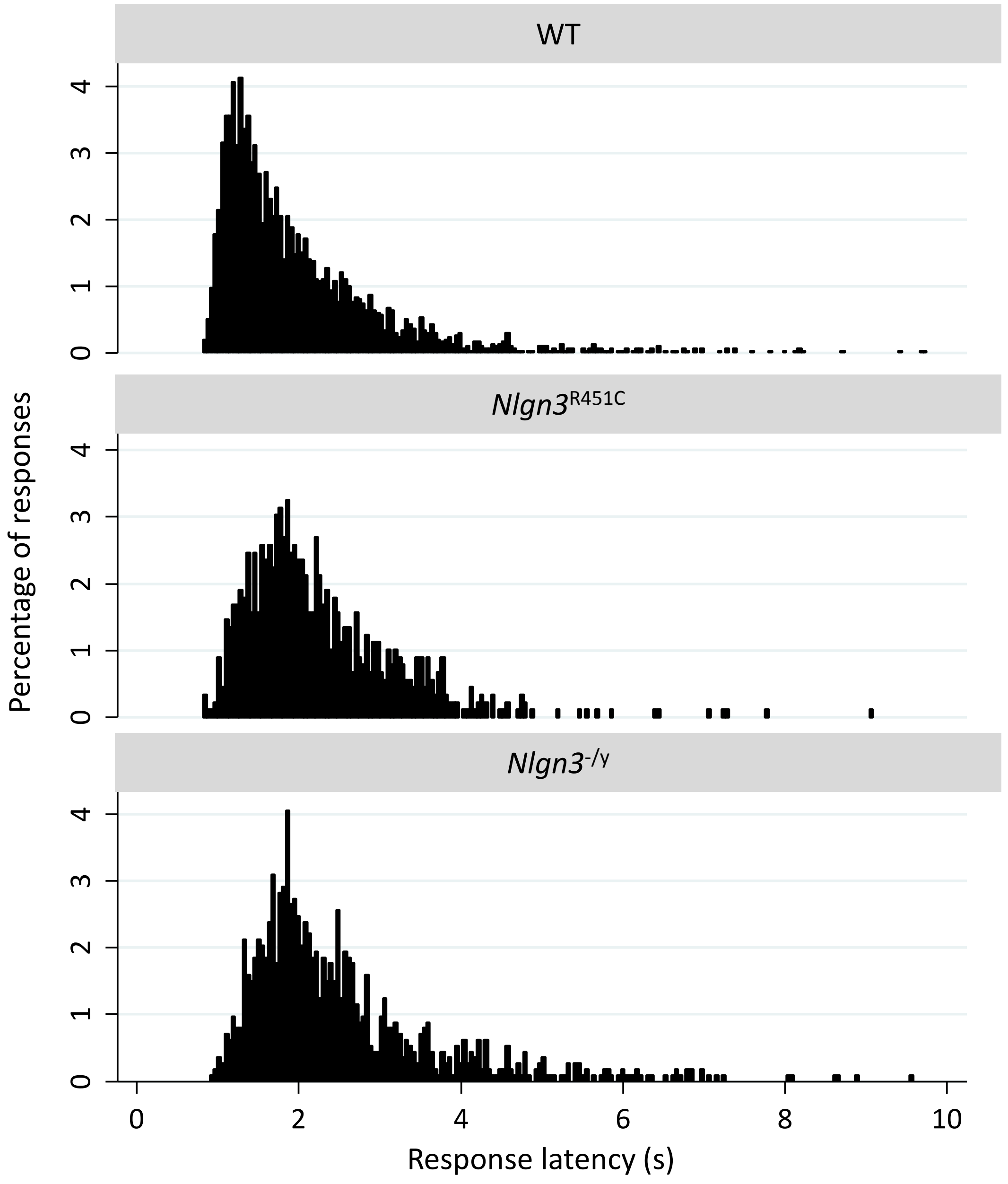

Supplementary Figure 3. Histogram of the skewed distribution of response latencies (with a long tail) during stimulus pair learning (Stage 2). Bars show percentage of incorrect response latencies in 200 bins between 0 – 10 seconds for pseudorandom first-presentation trials in pair $A^{+}B^{-}$.

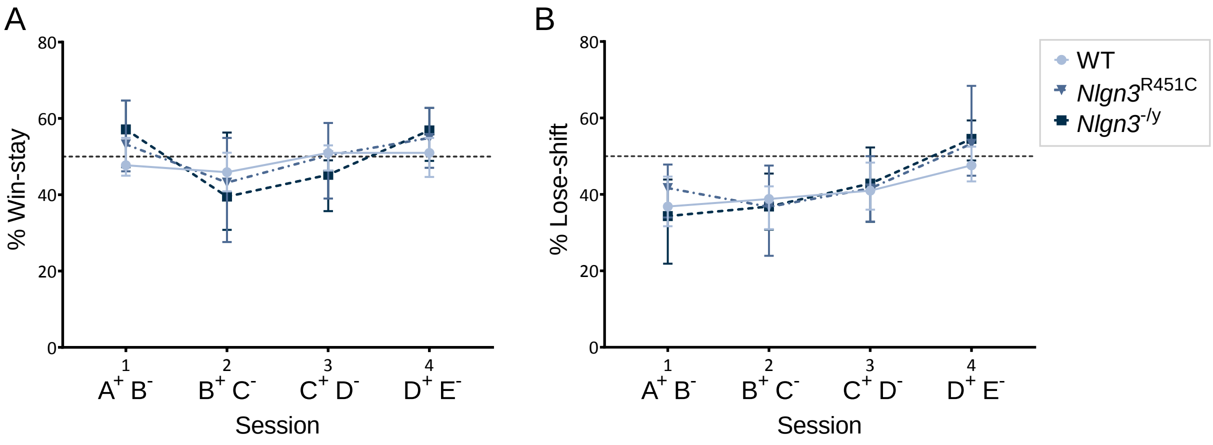

Supplementary Figure 4. Percentage of trials that were (A) Win-stay (win-stay/win-stay + win-shift) and (B) Lose-shift (lose-shift/lose-shift + lose-stay) for the 4 premise stimulus pairs across all genotypes during the first 4 sessions of training (Stage 1). For all genotypes, win-stay percentages remained at 50% chance level across sessions. In comparison, all mice were less likely than chance (<50%) during the first 3 sessions to shift their response on correction trials following incorrect responses, but the percentage of lose-shift trials gradually increased across sessions in Stage 1. Data presented as medians ± 95% confidence interval (CI).

METHOD	PARAMETERS	DESIGN	RESULT
EXPERIMENT 1<br>STEP 1:<br>STIMULUS PAIR LEARNING	5 stimulus pairs:<br>$A^{+}B^{-}$, $B^{+}C^{-}$, $C^{+}D^{-}$, $D^{+}E^{-}$, $E^{+}F^{-}$<br>Criterion = Average 30/40 (75%) correct across 2 sessions	Mixed-pair design of 1 session on each stimulus pair in order (i.e., 1 session on A+ B-, then one session on B+ C-, then C+ D-, then D+ E-, then E+ F-) then repeated. 40 trials/session; two sessions/day separated by >1hr rest.	No improvements in accuracy after 16 sessions of all 5 stimulus pairs.
EXPERIMENT 2<br>STEP 1:<br>STIMULUS PAIR LEARNING	5 stimulus pairs:<br>$A^{+}B^{-}$, $B^{+}C^{-}$, $C^{+}D^{-}$, $D^{+}E^{-}$, $E^{+}F^{-}$<br>Criterion = Average 30/40 (75%) correct across 2 sessions	Serial training design where each stimulus pair was trained to criterion before mice advanced to the next pair in the series.	Mice successfully acquired discrimination of all 5 stimulus pairs.
STEP 2:<br>INTEGRATION	5 stimulus pairs:<br>$A^{+}B^{-}$, $B^{+}C^{-}$, $C^{+}D^{-}$, $D^{+}E^{-}$, $E^{+}F^{-}$<br>No performance criterion	Serial integration (blocked): All 5 stimulus pairs within a session repeated in blocks of 4 (i.e. 4 trials of A+ B-, then 4 trials of B+ C-, and so on presented in order) and repeated twice.	Mice trained on this stage for a max. 5 sessions.
STEP 3:<br>INTEGRATION	5 stimulus pairs:<br>$A^{+}B^{-}$, $B^{+}C^{-}$, $C^{+}D^{-}$, $D^{+}E^{-}$, $E^{+}F^{-}$<br>Criterion = Average 30/40 (75%) correct across 2 sessions	Serial integration (single): All 5 stimulus pairs within a session repeated in order, then entire sequence repeated 8 times.	Trained until most mice completed 35 sessions but none reached criterion.
EXPERIMENT 3<br>STEP 1:<br>STIMULUS PAIR LEARNING	4 stimulus pairs:<br>$A^{+}B^{-}$, $B^{+}C^{-}$, $C^{+}D^{-}$, $D^{+}E^{-}$<br>Criterion = Average 30/40 (75%) correct across 2 sessions	Serial training design where each stimulus pair was trained to criterion before advancing mice to the next pair in the sequence.	Mice successfully acquired discrimination of all 4 stimulus pairs to criterion.
STEP 2:<br>SERIAL INTEGRATION	4 stimulus pairs:<br>$A^{+}B^{-}$, $B^{+}C^{-}$, $C^{+}D^{-}$, $D^{+}E^{-}$<br>Criterion of average 28/40 (70%) over 2 sessions or max. 30 sessions	Serial integration: Trials of all 4 stimulus pairs presented in serial order within a session and repeated; criterion adjusted for difficulty.	2/11 mice reached ≥70% criterion; 9/11 mice trained for 30 sessions before advancing.
STEP 3:<br>PSEUDORANDOM INTEGRATION	4 stimulus pairs:<br>$A^{+}B^{-}$, $B^{+}C^{-}$, $C^{+}D^{-}$, $D^{+}E^{-}$<br>Criterion of average 28/40 (70%) over 2 sessions or max. 20 sessions	Pseudorandom integration: Trials of all 4 stimulus pairs presented in pseudorandom order repeatedly within a session, criterion adjusted for difficulty.	3/11 mice reached ≥70% criterion; 8/11 mice trained for 20 sessions before advancing to TI test.
STEP 4:<br>TRANSTIVE INFERENCE TEST	4 stimulus pairs:<br>$A^{+}B^{-}$, $B^{+}C^{-}$, $C^{+}D^{-}$, $D^{+}E^{-}$<br>2 novel pairs: A>E, B>D<br>No performance criterion	Trials of 4 learned pairs (7 trials per pair) and 2 novel pairs (15 trials per pair) presented in pseudorandom order within a session.	Mice able to correctly discriminate A>E and B>D above chance accuracy.

Supplementary Table 1. Summary of optimisation Experiments 1-3 in C57BL/6J mice to refine the transitive inference touchscreen task.

	WT	*Nlgn3*^{R451C}	*Nlgn3*$^{-/y}$
Habituation	2.0 ± 0.0	2.0 ± 0.0	2.0 ± 0.0
Initial Touch	1.0 ± 0.0	2.0 ± 0.0	2.0 ± 0.0
Must Touch	1.0 ± 0.0	1.0 ± 0.0	1.0 ± 0.0
Must Initiate	1.1 ± 0.1	1.0 ± 0.0	1.0 ± 0.0
Punish Incorrect	2.2 ± 0.2	2.1 ± 0.2	2.0 ± 0.0

Supplementary Table 2. Sessions to criterion on pre-training stages to acquire operant learning. Data presented as mean ± SEM.

Relative to A^+B^-	WT				*Nlgn3*^{R451C}				*Nlgn3*$^{-/y}$			
	p > t	OR	95% CI		*p* > t	OR	95% CI		*p* > t	OR	95% CI	
**B^+C^-**	< 0.001	0.675	0. 635	0.717	< 0.001	0.536	0.485	0.592	< 0.001	0.571	0.522	0.625
**C^+D^-**	< 0.001	0.629	0.594	0.665	< 0.001	0.488	0.445	0.536	< 0.001	0.502	0.461	0.547
**D^+E^-**	< 0.001	0.745	0.704	0.788	< 0.001	0.601	0.548	0.660	< 0.001	0.582	0.535	0.633

Supplementary Table 3. Effect of stimulus pair on the likelihood of responding correctly to trials (pseudorandom first-presentation) relative to pair A^+B^- during premise pair learning (Stage 2). Mixed effect logistic regression with individual mice treated as random effects. Effect sizes presented as odds ratio (OR) ± 95% confidence interval (CI).

	WT				$Nlgn3^{R451C}$				$Nlgn3^{-/y}$			
	p > t	aMD	95% CI		*p* > t	aMD	95% CI		*p* > t	aMD	95% CI	
**A^+B^-**	< 0.001	-0.123	-0.171	-0.075	0.004	-0.126	-0.212	-0.041	< 0.001	-0.138	-0.190	-0.087
**B^+C^-**	< 0.001	-0.507	-0.647	-0.367	< 0.001	-0.382	-0.553	-0.210	< 0.001	-0.403	-0.514	-0.292
**C^+D^-**	< 0.001	-0.104	-0.140	-0.069	< 0.001	-0.111	-0.167	-0.055	< 0.001	-0.107	-0.156	-0.059
**D^+E^-**	< 0.001	-0.066	-0.094	-0.038	0.043	-0.031	-0.061	-0.001	0.001	-0.058	-0.090	-0.025

Supplementary Table 4. Effect of session on Perseveration Index (PI) for all genotypes across all pairs during premise pair learning (Stage 2). Median regression with individual mice treated as clusters. Effect sizes presented as the adjusted median difference (aMD) between groups ± 95% confidence interval (CI).

Relative to A^+B^-	WT				*Nlgn3*^{R451C}				*Nlgn3*$^{-/y}$			
	p > t	OR	95% CI		*p* > t	OR	95% CI		*p* > t	OR	95% CI	
**B^+C^-**	< 0.001	0.475	0. 445	0.507	< 0.001	0.386	0.339	0.438	< 0.001	0.523	0.471	0.580
**C^+D^-**	< 0.01	0.916	0.858	0.978	< 0.001	0.634	0.558	0.720	*0.200*	*0.934*	*0.841*	*1.037*
**D^+E^-**	< 0.001	1.235	1.155	1.322	*0.116*	*0.901*	*0.791*	*1.026*	< 0.001	1.397	1.252	1.558

Supplementary Table 5. Effect of stimulus pair on the likelihood of responding correctly to correction trials relative to pair A+ B- during premise pair learning (Stage 2). Mixed effect logistic regression with individual mice treated as random effects. Effect sizes presented as odds ratio (OR) $\pm$ 95% confidence interval (CI).

		WT	*Nlgn3*^{R451C}	*Nlgn3*$^{-/y}$
Stage 3 **Serial Integration**	Median	780	728	780
	95% CI	399 - 780	208 - 780	728 - 780
Stage 4 **Pseudorandom Integration**	Median	780	702	780
	95% CI	468 - 780	156 - 780	676 - 780

Supplementary Table 6. Trials to criterion on the serial (Stage 3) and pseudorandom (Stage 4) integration training stages. Summary data presented as median ± 95% confidence interval (CI).

Relative to D^+E^-	All mice			
	p > t	OR	95% CI	
**A^+B^-**	< 0.004	0. 652	0.488	0.872
**B^+C^-**	< 0.001	0.268	0.202	0.355
**C^+D^-**	< 0.001	0.516	0.387	0.687
Relative to A^+B^-				
**B^+C^-**	< 0.001	0.411	0.314	0.538
**C^+D^-**	0.087	0.791	0.605	1.034
**D^+E^-**	0.004	1.533	1.147	2.050

Supplementary Table 7. Likelihood of responding correctly on the 4 learned premise pairs (relative to A^+B^- or D^+E^-) on the transitive inference test. Mixed effect logistic regression with individual mice treated as random effects. Effect sizes presented as odds ratio (OR) ± 95% confidence interval (CI).

A

**CORRECT** Relative to WT	***Nlgn3*^R451C^**				***Nlgn3*^-/y^**			
	p > t	aMD	95% CI		*p* > t	aMD	95% CI	
**A⁺B⁻**	0.034	0.174	0.013	0.335	< 0.001	0.361	0.179	0.544
**B⁺C⁻**	0.029	0.407	0.043	0.771	*0.495*	*0.121*	*-0.227*	*0.469*
**C⁺D⁻**	*0.568*	*0.082*	*-0.120*	*0.363*	*0.273*	*-0.154*	*-0.431*	*0.122*
**D⁺E⁻**	*0.761*	*-0.034*	*-0.251*	*0.183*	*0.914*	*-0.011*	*-0.209*	*0.187*
**INCORRECT** Relative to WT	*p* > t	aMD	95% CI		*p* > t	aMD	95% CI	
**A⁺B⁻**	< 0.001	0.372	0.181	0.565	< 0.001	0.511	0.273	0.749
**B⁺C⁻**	*0.124*	*0.391*	*-0.107*	*0.888*	*0.270*	*0.225*	*-0.175*	*0.625*
**C⁺D⁻**	0.074	0.285	-0.028	0.598	*0.551*	*-0.091*	*-0.390*	*0.208*
**D⁺E⁻**	*0.463*	*0.084*	*-0.140*	*0.307*	*0.577*	*0.062*	*-0.156*	*0.281*

B

**CORRECT** Relative to A⁺B⁻	**WT**				***Nlgn3*^R451C^**				***Nlgn3*^-/y^**			
	p > t	aMD	95% CI		*p* > t	aMD	95% CI		*p* > t	aMD	95% CI	
**B⁺C⁻**	< 0.001	0.755	0.551	0.959	< 0.001	1.056	0.777	0.1.336	< 0.001	0.496	0.261	0.731
**C⁺D⁻**	< 0.001	0.576	0.355	0.797	< 0.001	0.437	0.232	0.642	*0.290*	*0.096*	*-0.082*	*0.274*
**D⁺E⁻**	< 0.001	0.306	0.177	0.435	0.008	0.174	0.044	0.303	*0.957*	*-0.005*	*-0.178*	*0.168*
**INCORRECT** Relative to A⁺B⁻	*p* > t	aMD	95% CI		*p* > t	aMD	95% CI		*p* > t	aMD	95% CI	
**B⁺C⁻**	< 0.001	0.783	0.496	1.070	< 0.001	0.777	0.406	1.147	< 0.001	0.474	0.191	0.758
**C⁺D⁻**	< 0.001	0.539	0.289	0.788	0.011	0.326	0.075	0.577	*0.326*	*-0.113*	*-0.339*	*0.113*
**D⁺E⁻**	< 0.001	0.273	0.132	0.415	*0.884*	*-0.014*	*-0.205*	*0.176*	0.050	-0.023	-0.042	0.000

Supplementary Table 8. Correct and incorrect response latencies to trials (pseudorandom first-presentation) during premise stimulus pair learning (Stage 2) (A) relative to WT and (B) stimulus pair A^+B^-. Median regression with individual mice treated as clusters. Effect sizes presented as the adjusted median difference (aMD) between groups ± 95% confidence interval (CI).

A

	Relative to A^+B^-				Relative to B^+C^-				Relative to D^+E^-			
	p > t	aMD	95% CI		*p* > t	aMD	95% CI		*p* > t	aMD	95% CI	
A^+B^-					< 0.001	-0.364	-0.451	-0.277	< 0.001	-0.579	-0.685	-0.473
B^+C^-	< 0.001	0.364	0.277	0.451					< 0.001	-0.215	-0.301	-0.129
C^+D^-	< 0.001	0.285	0.210	0.360	0.016	-0.079	-0.143	-0.015	< 0.001	-0.294	-0.378	-0.210
D^+E^-	< 0.001	0.579	0.473	0.685	< 0.001	0.215	0.129	0.301				

B

	Relative to A^+B^-				Relative to B^+C^-				Relative to D^+E^-			
	p > t	aMD	95% CI		*p* > t	aMD	95% CI		*p* > t	aMD	95% CI	
A^+B^-					0.004	-0.117	-0.197	-0.037	< 0.001	-0.476	-0.611	-0.341
B^+C^-	0.004	0.117	0.037	0.197					< 0.001	-0.359	-0.497	-0.221
C^+D^-	< 0.001	0.154	0.069	0.239	*0.391*	*0.037*	*-0.048*	*0.121*	< 0.001	-0.322	-0.450	-0.194
D^+E^-	< 0.001	0.476	0.341	0.611	< 0.001	0.359	0.221	0.497				

Supplementary Table 9. Differences between (A) Correct and (B) incorrect response latencies for the 4 premise stimulus pairs (relative to A^+B^-, B^+C^- or D^+E^-) for all genotypes during pseudorandom integration (Stage 4, last 5 sessions of training). Median regression with individual mice treated as clusters. Effect sizes presented as the adjusted median difference (aMD) between groups ± 95% confidence interval (CI).
